# Polygenic overlap and shared genetic loci between loneliness, severe mental disorders, and cardiovascular disease risk factors suggest shared molecular mechanisms

**DOI:** 10.1038/s41398-020-01142-4

**Published:** 2021-01-05

**Authors:** Linn Rødevand, Shahram Bahrami, Oleksandr Frei, Aihua Lin, Osman Gani, Alexey Shadrin, Olav B. Smeland, Kevin S. O’ Connell, Torbjørn Elvsåshagen, Adriano Winterton, Daniel S. Quintana, Guy F. L. Hindley, Maren C. F. Werner, Srdjan Djurovic, Anders M. Dale, Trine V. Lagerberg, Nils Eiel Steen, Ole A. Andreassen

**Affiliations:** 1 NORMENT, Centre for Mental Disorders Research, Division of Mental Health and Addiction, Oslo University Hospital, and Institute of Clinical Medicine, University of Oslo, Oslo, Norway; 2 Center for Bioinformatics, Department of Informatics, University of Oslo, 0316 Oslo, Norway; 3 Department of Neurology, Oslo University Hospital, Oslo, Norway; 4 Department of Psychology, University of Oslo, Oslo, Norway; 5 KG Jebsen Centre for Neurodevelopmental Disorders, University of Oslo, Oslo, Norway; 6 Institute of Psychiatry, Psychology and Neuroscience, King’s College London, 16 De Crespigny Park, London, SE5 8AB UK; 7 Department of Medical Genetics, Oslo University Hospital, Oslo, Norway; 8 NORMENT Centre, Department of Clinical Science, University of Bergen, Bergen, Norway; 9 Department of Radiology, University of California San Diego, La Jolla, CA 92093 USA; 10 Multimodal Imaging Laboratory, University of California San Diego, La Jolla, CA 92093 USA; 11 Department of Psychiatry, University of California San Diego, La Jolla, CA USA; 12 Department of Neurosciences, University of California San Diego, La Jolla, CA 92093 USA

**Keywords:** Clinical genetics, Schizophrenia

## Abstract

Clinical and epidemiological evidence suggest that loneliness is associated with severe mental disorders (SMDs) and increases the risk of cardiovascular disease (CVD). However, the mechanisms underlying the relationship between loneliness, SMDs, and CVD risk factors remain unknown. Here we explored overlapping genetic architecture and genetic loci shared between SMDs, loneliness, and CVD risk factors. We analyzed large independent genome-wide association study data on schizophrenia (SCZ), bipolar disorder (BD), major depression (MD), loneliness and CVD risk factors using bivariate causal mixture mode (MiXeR), which estimates the total amount of shared variants, and conditional false discovery rate to evaluate overlap in specific loci. We observed substantial genetic overlap between SMDs, loneliness and CVD risk factors, beyond genetic correlation. We identified 149 loci jointly associated with loneliness and SMDs (MD *n* = 67, SCZ *n* = 54, and BD *n* = 28), and 55 distinct loci jointly associated with loneliness and CVD risk factors. A total of 153 novel loneliness loci were found. Most of the shared loci possessed concordant effect directions, suggesting that genetic risk for loneliness may increase the risk of both SMDs and CVD. Functional analyses of the shared loci implicated biological processes related to the brain, metabolic processes, chromatin and immune system. Altogether, the study revealed polygenic overlap between loneliness, SMDs and CVD risk factors, providing new insights into their shared genetic architecture and common genetic mechanisms.

## Introduction

Patients with severe mental disorders (SMDs), including schizophrenia (SCZ), bipolar disorder (BD), and major depressive disorder (MDD), have 15–20 years reduced life span compared to the general population^1^. A major cause of the increased mortality is a high cardiovascular disease (CVD) risk^2,3^, and some of this CVD risk seems to be related to an unhealthy lifestyle, medication side-effects, and genetic susceptibility to CVD^4–6^. More recently, evidence has emerged implicating loneliness as a factor that may contribute to CVD comorbidity^7,8^. Loneliness is defined as a subjective discrepancy between the desired and achieved level of social relationships^9^. Loneliness is a considerable concern in Western societies, reportedly affecting more than a fifth of adults in the United States and the United Kingdom^10^. Feeling lonely has increasingly been recognized as an important health issue, as people who feel alone have increased risk for premature death and CVD morbidity, even after controlling for factors such as health-related behavior, age, gender, marital status, and depressive symptoms^11–15^. The influence of deficient social relationships on mortality is shown to be comparable with well-established risk factors such as smoking and exceeds the risk associated with obesity and hypertension^12^. Notably, during the coronavirus pandemic, social isolation is increasing across the globe, and the expected mental and physical health effects are large^16–18^.

Loneliness is a particular challenge for people with SMDs^19^. The annual rate of loneliness is ~2.3 times higher in SMDs than in the general population^20,21^, and loneliness is related to poorer quality of life, functioning and recovery^22–24^. Despite the high prevalence and adverse effects of loneliness in SMDs with vulnerability to CVD, little is known about the mechanisms underlying this association. Furthermore, development of interventions that reduce loneliness and comorbid CVD in SMDs is precluded by this limited understanding. Several factors might contribute to the co-occurrence of loneliness and CVD risk in SMDs, including unhealthy lifestyle, stigma, and stress activation^7,25–27^. Moreover, the phenotypic overlap raises an intriguing question: to what extent does a shared genetic architecture between SMDs, loneliness, and CVD risk factors drive the observed association?

SMDs are complex disorders, with heritability estimates of 0.6–0.8 for SCZ and BD^28^, and ~0.4 for MDD^29^. Despite their different clinical characteristics, there is a substantial genetic overlap between the disorders^30,31^. Recent genome-wide association studies (GWASs) have identified several genetic variants associated with the disorders^32–34^. GWASs have also reported loci associated with CVD risk factors, including body mass index (BMI)^35,36^, type 2 diabetes mellitus (T2D)^37^, total cholesterol (TC)^38^, high-density lipoprotein (HDL) cholesterol^38^, systolic blood pressure (SBP)^39^, diastolic blood pressure (DBP)^39^, along with coronary artery disease (CAD)^40^. While loneliness is influenced by social network, support, and poverty^41,42^, its estimated heritability is 0.4–0.5^43^. Specific genetic determinants of loneliness were also recently identified^44^, and loneliness showed genetic correlation with MDD, SCZ, and body size^44^. However, the genetic correlations with SCZ and body size were low (*r*_g_ = 0.17) and insignificant with BD. A limitation with measures of genetic correlation is that the method requires consistent effect directions among the shared variants^45^. Thus, insignificant or low genetic correlations do not necessary imply no genetic overlap, but may rather be due to a mixture of positive and negative effect directions of the overlapping variants. Therefore, to obtain a comprehensive understanding of the genetic relationship between loneliness, SMDs and CVD risk, measures of genetic correlations should be complemented by tools that allow for the discovery of shared variants regardless of their effect directions^46^.

In the current study, we aimed to identify the shared genetic architecture of loneliness, SMDs and CVD risk factors beyond genetic correlations by applying the recently developed bivariate causal mixture model (MiXeR), which evaluates overlap at the architecture level, estimating the total number of shared and trait-specific genetic variants^47^. The results are presented with Venn diagrams visualizing the estimated shared and unique polygenic variants^47^. Further, we applied the conditional false discovery rate (condFDR) approach, which can uncover overlapping genetic variants irrespective of direction of effects. This method builds on an empirical Bayesian statistical framework, and increases the power to detect shared loci by leveraging the combined power of several large independent GWASs^48–50^. We have used this approach to identify the shared genetic underpinnings of several complex human traits and disorders in recent years^5,6,51^. This method fits well to disentangle any complex genetic relationship with loneliness, SMDs and CVD risk factors.

Here we investigated the genetic relationship between SMDs, loneliness, and CVD risk by analyzing summary data from recent large-scale GWASs using MiXeR^47^ and condFDR^50^. We hypothesize that genetic determinants contributing to SMDs and comorbid CVD, overlap with the genetic risk for loneliness, with different levels of overlap across SCZ, BD, and MDD given their different clinical characteristics. Investigating overlap in genetic variants can elucidate important shared pathobiology and have implications for the understanding of CVD comorbidity in SMDs.

## Methods

### Participant samples

We obtained GWAS summary data on SCZ (*n* = 82,315), BD (*n* = 51,710), and major depression (MD) (*n* = 450,619) from Psychiatric Genomics Consortium^32–34^. We use MD instead of the diagnostic term “major depressive disorder”, since many of the MD cases were identified by self-report^33^. Data on loneliness (*n* = 452,302) were obtained from the UK Biobank study based on self-reported responses to three questions regarding perceived loneliness, frequency of social contact, and the ability to confide in someone close^44^. The vast majority of participants in the UK Biobank are healthy individuals. A small fraction of participants has a psychiatric diagnosis, including 2483 with SCZ, 2123 with BD and 8276 with MD (UK Biobank data field 41270, Supplementary Methods and Supplementary Table 1). While the number of participants with self-reported depression is higher (Supplementary Table 2)^52^, Day et al.^44^ performed a sensitivity analysis by repeating the loneliness GWAS excluding individuals with self-reported depression (*N* = 26,801), which did not result in any appreciable change in results^44^. Consequently, it seems unlikely that psychiatric diagnoses that are far less prevalent than self-reported depression in the UK Biobank (see Supplementary Tables 1, 2), have confounded the results significantly. Therefore, similar to Day et al.^44^ we did not exclude participants with self-reported depression or other psychiatric diagnoses from the loneliness GWAS data set. Further, we used GWAS data on the CVD risk factors BMI, TC, SBP, DBL, HDL-C, and T2D, (*n* = 159,208–795,640 depending on CVD risk factor)^35–39^. We also included CAD (*n* = 185,000) as this is a major CVD^40^, and smoking for supplementary analysis^53^. For cond/conjFDR analyses, overlapping cohorts between GWAS samples were excluded. For details, see Supplementary Methods and original publications^32–40,44,53^. All GWASs investigated in the current study were approved by local ethics committees, and all participants provided informed consent^32–40,44,53^. The Regional Committee for Medical Research Ethics—South-East Norway has evaluated the current protocol and found that no additional institutional review board approval was necessary because no individual data were used.

### Statistical analysis

For further information about the statistical approaches described below, see Supplementary Methods. We explored pleotropic enrichment by constructing conditional quantile–quantile (Q–Q) plots. Enrichment is visualized in conditional Q–Q plots as successive leftward deflections from the null distribution^48,49,54^.

We used the statistical tool, MiXeR, which quantifies polygenic overlap irrespective of genetic correlation using GWAS summary statistics^47^. This method estimates the total number of shared and trait-specific causal variants (i.e., variants with nonzero additive genetic effects on a trait). We applied MiXeR for phenotypes that demonstrated most significant genetic overlap in conditional Q–Q plots (i.e., loneliness and SMDs and BMI). To evaluate model fit, i.e., the ability of the MiXeR model to predict the actual GWAS data, we constructed modeled vs. actual conditional Q–Q plots, log-likelihood plot, and Akaike information criterion (AIC). For further information about MiXeR, see Supplementary Methods and Frei et al.^47^.

To improve the discovery of specific genetic variants shared between phenotypes, we applied the condFDR statistical framework^48,49^. This approach is an extension of the standard FDR method, and re-ranks the test statistics of a primary phenotypes (e.g., SCZ) based on the strength of the association with a secondary phenotype (e.g., loneliness)^48,49,54^. After repeating the condFDR analysis for both phenotypes, we identified *shared* genetic loci at conjunctional FDR (conjFDR) <0.05^48,54^. The conjFDR is defined as the maximum of two condFDR values, which provides a conservative estimate of the FDR for association with both phenotypes^48,54^. Unlike MiXeR, conjFDR identifies the localization of specific shared variants^48,54^. Thus, MiXeR and conjFDR are complementary methods that offer information about genetic overlap on different levels (i.e., *total amount* of overlap and *specific* shared variants, respectively). These methods do not build on one another; rather, conjFDR is an extension of condFDR. Thus, we applied conjFDR for phenotypes that demonstrate polygenic overlap based on condFDR analysis, and applied condFDR for phenotypes that showed polygenic overlap in conditional Q–Q plots.

### Genomic loci definition and effect direction

We defined independent genomic loci using FUMA (http://fuma.ctglab.nl/ and Supplementary Methods)^55^. Further, we evaluated the directional effects of the loci shared between loneliness and SMDs and CVD risk factors by comparing their *z-*scores or odds ratios. Effect direction could not be computed for blood pressure because effect scores were not available from the original GWAS^39^. Genetic correlations were estimated using MiXeR and LD score regression^47,56^.

### Functional annotation

We used FUMA^55^ to functionally annotate candidate SNPs within the genomic loci with a condFDR or conjFDR value of <0.10 and an LD *r*^2^ ≧ 0.6 with one of the independent significant SNPs. We further annotated SNPs using three tools: *Combined Annotation Dependent Depletion*^57^, which predicts the deleteriousness of SNPs on protein structure/function; *RegulomeDB*^58^, which predicts regulatory functions; and *chromatin states* that indicates the transcription/regulation effects of chromatin states at the SNP locus^59,60^. We also used FUMA^55^ to map lead and candidate SNPs to genes and investigate whether these genes were overrepresented in gene-sets associated with particular biological functions (Supplementary Methods).

## Results

### Genetic overlap between SMDs and loneliness

In conditional Q–Q plots, we observed SNP enrichment for loneliness as a function of the significance of SNP associations with MD, SCZ, and BD (Supplementary Fig. 1). This indicates polygenic overlap between the phenotypes. The reverse conditional Q–Q plots also demonstrate consistent enrichment in MD, SCZ, and BD given associations with loneliness (Supplementary Fig. 2).

**Fig. 1:**
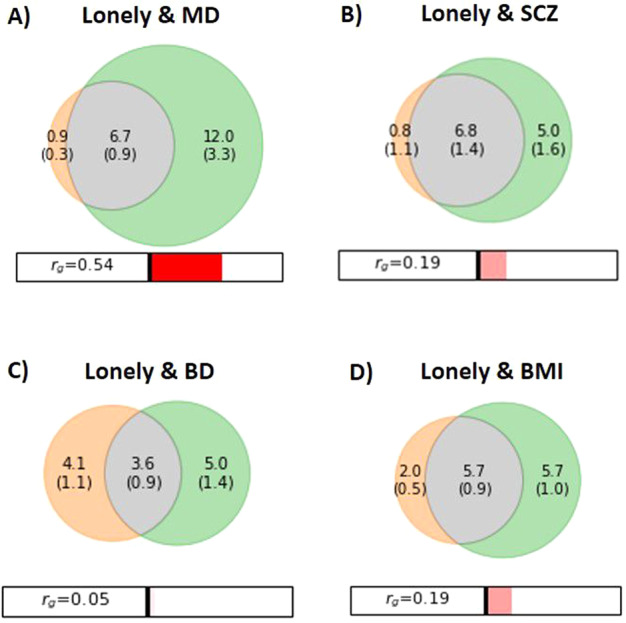
Venn diagrams of unique and shared polygenic variants. Venn diagrams showing polygenic overlap (gray) between **A**) loneliness (orange) and major depression (MD) (green), **B**) schizophrenia (SCZ) (green), **C**) bipolar disorder (BD) (green), and **D**) body mass index (BMI) (green). The numbers indicate the estimated quantity of causal variants (in thousands) per component, explaining 90% of SNP heritability in each phenotype, followed by the standard error. The size of the circles reflects the degree of polygenicity. Figures generated from MiXeR.

We performed MiXeR analysis with loneliness and the three SMDS after observing their polygenic overlap in Q–Q plots. Using MiXeR we found further evidence of polygenic overlap between loneliness and SMDs (Fig. 1; Supplementary Figs. 3,5). The Venn diagram for loneliness and MD demonstrates substantial polygenic overlap, sharing 6.7K out of 19.6K causal variants (Fig. 1A). Further, loneliness and SCZ also show polygenic overlap, sharing 6.8K out of 12.6K causal variants (Fig. 1B). In addition, loneliness and BD exhibit polygenic overlap, sharing 3.6K out of 12.7K causal variants (Fig. 1C). The MiXeR estimates adequately model the GWAS data (Supplementary Figs. 3–5; Supplementary Results), while the results of loneliness vs. MD analysis are more uncertain. Negative AIC values indicate that the MiXeR model cannot be adequately differentiated from a scenario of maximum possible overlap and a scenario of minimum overlap (Supplementary Table 3). A larger MD GWAS is needed to obtain more certain MiXeR estimates.

MiXeR estimates of genetic correlation (Fig. 1A–C) were consistent with those of LD score regression (Table 1). Loneliness exhibited a significant positive genetic correlation with MD and weaker, yet significant, correlation with SCZ, but not with BD (Table 1).

**Table 1:** Shared loci between loneliness and SMDs and CVD risk factors.

Associated phenotype	Shared conjFDR	Loci (*n*) concordant effect (%)	Genetic correlation
SMD			
MD	67	95.5%	**0.570** (*p* = 2.74E−116)
SCZ	54	74.1%	**0.167** (*p* = 5.08E–12)
BD	28	61.7%	0.018 (*p* = 0.60)
CVD risk factor			
BMI	36	69.4%	**0.182** (*p* = 3.73E−17)
TC	6	83.3%	0.039 (*p* = 0.26)
HDL-C	5	60.0%	−**0.101** (*p* = 6.62E−5)
SBP	9	na	na
DBP	4	na	na
T2D	1	na	**0.119** (*p* = 0.0003)
CAD	12	58.3%	**0.129** (*p* = 4.60E−5)
Smoking	0	na	**0.252** (*p* = 0.0002)

Number of shared loci at conjFDR <0.05, concordant effect directions in percentage, and genetic correlation estimated by LD score regression. Bold values in the genetic correlation column are significant after Bonferroni correction (*p* < 0.05/11).

*SMD* severe mental disorder, *MD* major depression, *SCZ* schizophrenia, *BD* bipolar disorder, *CVD* cardiovascular disease, *BMI* body mass index, *TC* total cholesterol, *HDL-C* high-density lipoprotein cholesterol, *SBP* systolic blood pressure, *DBP* diastolic blood pressure, *T2D* type 2 diabetes mellitus, *CAD* coronary heart disease, *na* not available, *conjFDR* conjunctional FDR, *Na* effect directions not available from the SBP/DBP GWAS. As there were no shared loci between loneliness and smoking, percentage of concordant effects were not computed. As only one shared locus was found between T2D and loneliness, percentage with concordant effect is not given.

Further, using condFDR analysis, we discovered several SNPs significantly associated with loneliness conditional on their association with MD, SCZ and BD (Supplementary Tables 4–6), and vice versa (Supplementary Table 7) at condFDR <0.01.

### Genetic overlap between CVD risk factors and loneliness

We uncovered polygenic overlap between loneliness and CVD risk factors. In the conditional Q–Q plots, we observed SNP enrichment for loneliness as a function of the significance of the association with CVD risk factors (Supplementary Fig. 6), and vice versa (Supplementary Fig. 7), suggesting polygenic overlap between loneliness and CVD risk factors, especially BMI.

MiXeR was performed with loneliness and BMI given their substantial polygenic overlap demonstrated by conditional Q–Q plot. MiXeR revealed considerable polygenic overlap between loneliness and BMI, sharing 5.7K out of 13.4K causal variants (Fig. 1D). MiXeR results for BMI should be interpreted with caution due to more uncertain estimates (Supplementary Fig. 8; Supplementary Table 3).

MiXeR estimates of genetic correlation (Fig. 1D) were consistent with those of LD score regression (Table 1). Loneliness showed significant positive genetic correlations with BMI, smoking, CAD and T2D, and negative genetic correlation with HDL-C (Table 1).

Further, using condFDR, we identified several loneliness SNPs conditional on their association with CVD risk factors (Supplementary Tables 8–15), and vice versa (Supplementary Table 16) at condFDR <0.01.

### Genetic loci shared between SMDs, loneliness, and CVD risk factors

**Fig. 2:**
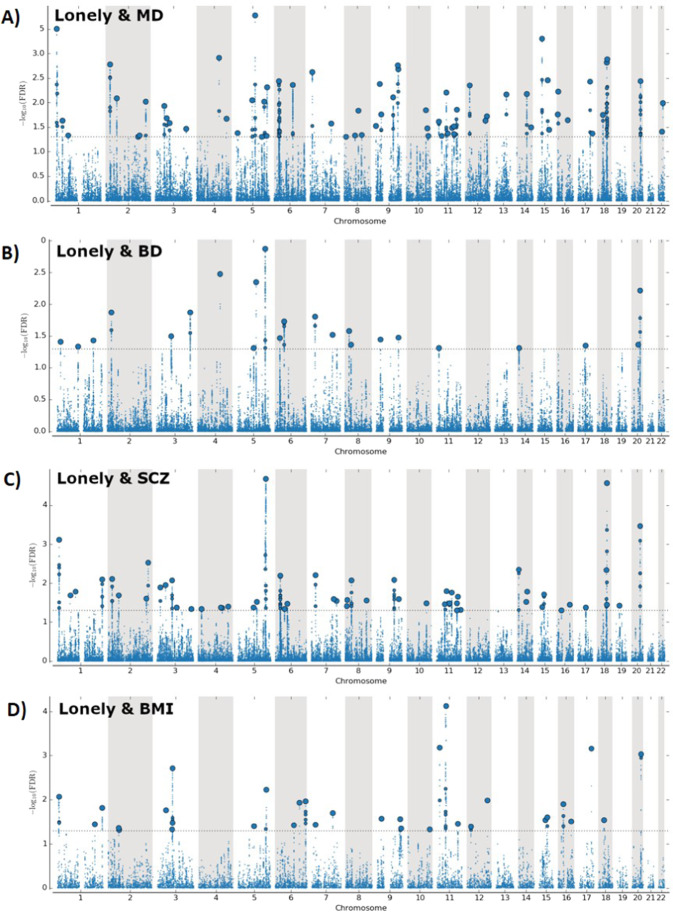
Common genetic variants jointly associated with loneliness and MD, BD, SCZ, and BMI at conjFDR <0.05. Manhattan plots for loneliness and **A**) major depression, **B**) bipolar disorder, **C**) schizophrenia and **D**) body mass index. Manhattan plots showing the −log10 transformed conjFDR values for each SNP on the *y* axis and chromosomal positions along the *x* axis. SNPs with conjunction FDR < 0.05 (i.e., −log10 FDR > 1.3) are shown with enlarged data points. A black circle around the enlarged data points indicates the most significant SNP in each LD block. The figure shows the localization of the “conjunctional loci”, and further details are provided in Supplementary Tables 17–20. MD major depression, SCZ schizophrenia, BD bipolar disorder, BMI body mass index, conjFDR conjunctional FDR.

At conjFDR <0.05, loneliness shared 67 loci with MD, 54 loci with SCZ, and 28 loci with BD (Fig. 2A–C, Table 1; Supplementary Tables 17–19). Some of these were overlapping between the SMDs (27), yielding a total of 122 distinct loci associated with both loneliness and SMDs. Among these shared loci, 115 loci were not identified in the original loneliness GWAS^44^. We evaluated the directionality of allelic effects in the loci shared between the phenotypes by investigating their *z*-scores. As denoted by the sign of the effect sizes, effect directions were mostly consistent (Table 1; Supplementary Tables 17–19). The majority of MD risk alleles (95.5%), SCZ risk alleles (74.1%), and BD risk alleles (61.7%) showed same effect direction in loneliness (Supplementary Tables 17–19).

In addition, loneliness shared multiple loci with CVD risk factors, including BMI (36 loci; Fig. 2D), TC (6 loci), HDL-C (5 loci), SBP (9 loci), DBP (4 loci), CAD (12 loci), and T2D (1 locus) (Table 1; Supplementary Tables 20–26; Supplementary Fig. 9), but no loci shared with smoking. Some of these were overlapping across CVD risk factors, yielding a total of 55 distinct loci shared between loneliness and CVD risk factors. Among these shared loci, 49 were not identified in the original loneliness GWAS^44^. For the loci shared between loneliness and CVD risk factors, we discovered same effect directions of 69.4% of loci shared with BMI, 83.3% of loci shared with TC, 60% of loci shared with HDL-C, and 58.3% of loci shared with CAD (Supplementary Tables 20–26).

**Fig. 3:**
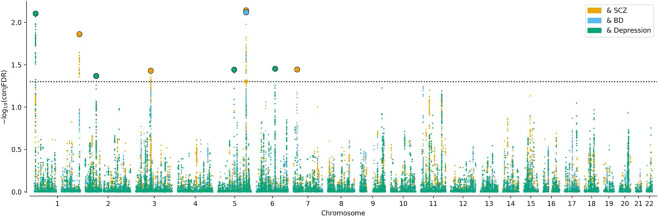
Common genetic variants jointly associated with SMDs, BMI and loneliness at conjFDR <0.05. Manhattan plot showing the −log10 transformed conjFDR values for each SNP on the *y* axis and chromosomal positions along the *x* axis. SNPs with conjunction FDR < 0.05 (i.e., −log10 FDR > 1.3) are shown with enlarged data points. A black circle around the enlarged data points indicates the most significant SNP in each LD block. The figure shows the localization of the “conjunctional loci”. SMDs severe mental disorders, MD major depression, SCZ schizophrenia, BD bipolar disorder, BMI body mass index, conjFDR conjunctional FDR.

Further, the trio conjFDR analyses identified loci shared between loneliness, BMI and MD (4), SCZ (5), and BD (1) (Fig. 3; Supplementary Tables 27–29). 60% of the loci shared between both loneliness, SMDs, and BMI possessed same effect directions (Supplementary Tables 27–29). Further, genetic correlations were in line with the consistent effect directions (Table 1).

Altogether, we identified a total number of 163 distinct loci shared between loneliness, SMDs, and CVD risk factors. Of these shared loci, 153 were not identified in the original loneliness GWAS^44^. To visualize the shared loci, we constructed conjFDR Manhattan plots (Figs. 2, 3; Supplementary Fig. 9) where all SNPs without pruning are shown, and the independent lead SNPs are encircled in black.

### Functional annotation

Functional annotation of all SNPs with a conjFDR value <0.1 within loci shared between loneliness and either SMDs or CVD risk factors demonstrated that these were mostly intronic and intergenic (Supplementary Tables 30–39). Gene-mapping of shared variants between loneliness and SMDs and CVD risk factors implicated brain-expressed genes (Supplementary Tables 30–39; Supplementary Results). For gene-set analyses, we focused on genes mapped to the loci shared between loneliness and SMDs and BMI, as these phenotypes showed most genetic overlap in the above results. Gene-set analyses for loneliness and SMDs discovered several biological processes, including “chromatin assembly”, “negative regulation of biosynthetic process”, “immune system development”, “synapse”, and “dentritic tree” (Supplementary Tables 40–42; Supplementary Results). Gene-set analyses for loneliness and BMI implicated “positive regulation of biosynthetic process” and “regulation of response to cytokine stimulus” (Supplementary Table 43; Supplementary Results). Further information about FUMA results are provided in Supplementary Results and Supplementary Tables 17–43.

## Discussion

Here, we discovered polygenic overlap between loneliness, SMDs and CVD risk factors and quantified their shared genetic architecture. We identified shared loci between loneliness and MD (67 loci), SCZ (54 loci) and BD (28 loci), and loneliness and CVD risk factors (55 loci). In addition, 10 loci were found to jointly influence SMDs, loneliness and BMI. Among the shared loci identified, 153 were novel to loneliness. While there was distinct differences between MD, SCZ, and BD, the majority of the shared variants (~80%) showed consistent effect directions, suggesting that genetic susceptibility to loneliness may also increase the risk of SMDs and CVD. The present results, together with prior evidence of genetic overlap between SMDs and CVD risk factors^5,6^, demonstrate shared genetic loci between loneliness, SMDs, and CVD risk factors, which may underlie some of the clinical relationship between loneliness, SMDs, and CVD comorbidity.

We used MiXeR^47^ to reveal polygenic overlap between loneliness, SMDs and BMI irrespective of genetic correlation. We applied conjFDR to leverage the boost in power from cross-trait enrichment, and uncovered multiple shared genetic variants between loneliness, SMDs and CVD risk factors. The conjFDR approach extends measures of genetic correlation by allowing discovery of shared loci regardless of their effect directionality^48,54^. Most of the loci shared between loneliness and MD (95.5%) and SCZ (74.1%) had the same effect direction, confirming the positive genetic correlation^44^. However, many of the shared loci between BD and loneliness had mixed effect directions, in line with the non-significant genetic correlation^44^. This demonstrates the usefulness of the condFDR approach to discover polygenic overlap between complex phenotypes despite the lack of genetic correlation. The results indicate that large fractions of the genomic risk architectures underlying MD, SCZ, and BD also influence loneliness, albeit in a different manner, providing new insights into their genetic nature.

Genetic risk factors of loneliness involve a propensity to experience psychological pain in response to social disconnection^26^. The perception of being socially disconnected introduces a hypervigilance to social threats, which can cause cognitive biases:^26^ lonely individuals appear to perceive the social world as more threatening, and expect and remember more negative social experiences (e.g., rejection)^25,26^. Although speculative, negative social expectations may increase the risk of paranoia, and thereby, the risk of developing a psychotic disorder^61,62^. Therefore, a genetic overlap between loneliness and SCZ may reflect shared genetics influencing a tendency to view the world as unsafe, contributing to poor social interactions and, thereby, increase the risk of loneliness and psychotic disorders. Also, loneliness is likely to be associated with social withdrawal and amotivation, which are negative symptoms in SCZ. Further, cognitive biases tend to negatively influence the behavior of lonely individuals (e.g., exhibit less interest and trust) which may discourage others from seeking contact and elicit depressive symptoms^25,26^. In addition, loneliness is associated with difficulties regulating emotions^63^, including diminished ability to downregulate negative emotions, similar to what is seen in MD^64^. Accordingly, the genetic overlap between loneliness and MD may reflect a genetic disposition to cognitive biases, emotional dysregulation, and behavior patterns (e.g., social withdrawal).

Similar processes may be involved in BD, which is characterized by mood disturbances, with psychotic features in 60%^65^. We may speculate that people with BD who exhibit psychotic symptoms like paranoia, are more prone to social withdrawal, contributing to loneliness. Conversely, individuals who are more socially active in manic phases, may feel less lonely. However, uncritical social behavior related to mania may contribute to social rejection and thus induce loneliness. We need further research on loneliness across different types of mood episodes in BD, which so far has provided inconsistent results^23,66^. The phenotypic heterogeneity in BD would be in line with our findings of many loci with mixed effect directions in BD and loneliness, while no genetic correlation. Taken together, our findings suggest that genetic determinants of mental processes and behavior contributing to loneliness overlap with SMDs. However, environmental factors such as a limited social network, lack of opportunities for social interactions, poverty and stigma^27,67^, remain important predictors of loneliness in SMDs.

Some of the genetic overlap discovered between loneliness and MD may be due to loneliness being an aspect of the phenomenology of MD. Still, considerable evidence suggests that depression and loneliness are distinct; while loneliness is a negative feeling signaling inadequate social contact, depression is a psychiatric diagnosis reflecting a more general dysphoric state^25,26,68^. A distinction between loneliness and depression is also supported by a loneliness GWAS that demonstrated that while loneliness and MD are genetically correlated, the loneliness loci remained significant after excluding individuals with depression from the analyses^44^.

By using conjFDR we discovered 55 loci that jointly influence loneliness and CVD risk factors. Further, we found overlapping loci between both loneliness, SMDs and BMI. The majority of the shared SNPs possessed the same effect directions, in line with positive genetic correlations identified between loneliness, CAD and most CVD risk factors. These findings imply that genetic susceptibility to loneliness is related to increased CVD risk, consistent with epidemiological data of positive associations between loneliness and CVD risk^11–15,69,70^. Several potential mechanisms may link loneliness to CVD risk^25^, including stress activation, lifestyle and psychological coping^25^. In particular, loneliness has been linked to activation of the hypothalamic–pituitary–adrenal axis^71^, which in turn has been implicated in the development of atherosclerosis^72^. Loneliness may also have indirect effects on CVD through lifestyle^25^, emotional regulation^25^, and mental illness^13^. Our findings suggest that the co-occurrence of loneliness and CVD risk may partly be driven by shared genetic architecture, and may explain some of comorbid CVD in SMDs. Further, gene-set analyses of the shared loci between loneliness and SMDs indicated genes associated with biological processes involving chromatin processes and brain functions, including synapses and dendrites. This provides plausible genetic links between loneliness, SMDs, and brain function. The gene-set analyses of loneliness loci shared with SMDs and BMI also indicated genes related to metabolic mechanisms and immune system, which have been implicated in the pathophysiology of SMDs and CVD morbidity^73^. However, experimental investigations are necessary to understand how the identified variants influence brain, metabolic and immune system development and function. Further, gene-mapping of shared variants between loneliness and SMDs and CVD risk factors, implicated genes expressed in brain tissue. Although the identified genes are not necessarily the genes by which the genetic variants exert their phenotypic effect, the results support the importance of brain-expressed genes in the shared genetic etiology of SMDs, loneliness and CVD. Thus, it seems likely that the shared genetic variants, together with environmental factors, contribute to brain dysfunction that affect different mental processes (cognitive bias, emotional regulation) and behavior (e.g., lifestyle, withdrawal) and thereby associated with the development of SMDs, loneliness and CVD. Other pathways are also possible; for instance, shared variants between loneliness and BMI may affect metabolism and increase the risk of overweight, which may hamper self-esteem contributing to development of loneliness and SMDs.

Although loneliness is highly prevalent in SMDs and associated with poorer quality of life, lower functioning and higher CVD risk^7,8,22–24^, interventions that effectively reduce loneliness in people with SMDs are limited^67^. Promising results suggest that correcting maladaptive social thinking offers a chance for reducing loneliness in people with mental disorders^74^. Our findings highlight the importance of an integrated approach to people with SMDs focusing on social contact. The results are also relevant for the social isolation strategies to prevent the coronavirus pandemic: While social distancing may protect against the coronavirus infection, it may increase loneliness^16–18^. Our findings suggest that people with SMDs may have a genetic susceptibility for loneliness, making them particularly vulnerable to these adverse effects of the solitude enforced in numerous countries^16–18^. Preventing and reducing loneliness may have beneficial effects both on psychosocial functioning, quality of life, and the illness course itself. Whether reducing loneliness in SMDs may also improve cardiovascular health, should be explored in future research.

Loneliness is a complex phenotype characterized by the perception that one’s social needs are not being met^9,25^. A challenge in studying loneliness has been the lack of a measure suitable for large-scale studies^75^. Therefore, recommendations for loneliness assessment in large studies were recently published^76^. A direction question of loneliness is recommended at a minimum^76^, such as “Do you often feel lonely?” used in the UK Biobank^44^. In addition, indirect measures of loneliness are recommended as loneliness is associated with stigma and, therefore, some people may be reluctant to admit to feeling lonely^75,76^. While the UK Biobank did not use any of the proposed indirect items^76^, participants were asked about their ability to confide in someone close^44^. Lonely people perceive themselves as less able to confide and have fewer people to confide in than non-lonely individual^77,78^, providing support for this item as an indirect probe of loneliness. Further, to increase power, the loneliness GWAS also included data on frequency of contact with family and friends and living alone^44^. This data concerns information about objective rather than subjective social isolation. However, lonely people tend to spend more time alone^79^ and are more likely to live alone than people who are not lonely^80^. Further support for the association between loneliness and objective isolation comes from the loneliness GWAS: the genetic loci identified in the complete analysis (including perceived loneliness, ability to confide, frequency of contact and living alone) were similar to those reported from analyzing only subjective loneliness^44^. Nevertheless, loneliness and objective isolation are distinct, and the loneliness measure in the UK biobank is limited by not using the best validated loneliness items^75,76^.

In conclusion, our study demonstrates shared genetic loci between loneliness, SMDs, and CVD risk factors, providing new insights into their shared genetic architecture. This suggests a potential genetic basis for the clinical association between loneliness, SMDs, and CVD. The findings further our understanding of comorbid CVD in SMDs and, ultimately, may form the basis of prevention and treatment development. The study illustrates the utility of the condFDR approach to increase gene discovery and disentangle the complex genetic relationship between loneliness, SMDs, and CVD risk factors.

## Supplementary information

Supplementary tables

Supplementary information

## Data Availability

The datasets analyzed during the current study are available in repositories of GWASs: SCZ: https://www.med.unc.edu/pgc/download-results/scz/. BD: https://www.med.unc.edu/pgc/download-results/bip/. MD: https://www.med.unc.edu/pgc/download-results/mdd/. Loneliness: https://www.repository.cam.ac.uk/handle/1810/277812. BMI: https://portals.broadinstitute.org/collaboration/giant/index.php/GIANT_consortium_data_files. TC: http://csg.sph.umich.edu/abecasis/public/lipids2013/. SBP: http://www.georgehretlab.org/. DBP: http://www.georgehretlab.org/. HDL-C: http://csg.sph.umich.edu/abecasis/public/lipids2013/. T2D: https://diagram-consortium.org/downloads.html. CAD: http://www.cardiogramplusc4d.org/data-downloads/. Smoking: https://www.med.unc.edu/pgc/download-results/tag/. All data/results generated during the current study are included in this published article [and its Supplementary Information Files]. Supplementary Information is available at the journal’s website.

## Code availability

Codes used for carrying out the described analyses are available here: https://github.com/precimed/pleiofdr, https://github.com/precimed/mixer, https://github.com/bulik/ldsc.
