# Supplementary material for: Polygenic overlap and shared genetic loci between loneliness, severe mental disorders, and cardiovascular disease risk factors suggest shared molecular mechanisms
Source: Transl Psychiatry. 2021 Jan 5;11:3. doi: 10.1038/s41398-020-01142-4 (PMC7790035; doi:10.1038/s41398-020-01142-4)
Supplement: Supplementary file 2 — Supplementary information [file 41398_2020_1142_MOESM2_ESM.docx]

**SUPPLEMENTARY INFORMATION for**

**Polygenic overlap and shared genetic loci between loneliness, severe mental disorders and cardiovascular disease risk factors suggest shared molecular mechanisms**

**SUPPLEMENTARY METHODS**

**Participant samples**

We obtained GWAS results in the form of summary statistics. Data on schizophrenia (SCZ), bipolar disorder (BD) and major depression (MD) were retrieved from Psychiatric Genomics Consortium (PGC)^1-3^. The SCZ dataset contained 49 non-overlapping case-control samples (34 241 cases with SCZ or schizoaffective disorder and 45 604 controls) and 3 family-based association studies (1235 parent affected-offspring trios)^1^. The BD dataset consisted of 20 352 cases and 31 358 controls from 32 samples^2^. Among the cases, 14,879 individuals were diagnosed with BD type I (BD1), 3,421 with BD type II (BD2), 977 with schizoaffective disorder, bipolar type (SAB), and the remaining unspecified BD^2^. The major depression (MD) dataset involved 135 458 cases and 344 901 controls^3^. The UK biobank cohort (n = 29 740) was excluded from the MD data set to avoid sample overlap.

Data on loneliness was obtained from the general population in the UK Biobank study (n = 452 302)^5^. 487 647 people responded to three questions regarding perceived loneliness, frequency of social contact, and ability to confide in someone close^5^. Genomic and phenotypic data were available from 452 302 participants after quality control criteria. The UK Biobank primarily compromises healthy individuals, and a small proportion of participants have a psychiatric diagnosis based on the International Classification of Diseases (ICD)-10, retrieved from health records (UK Biobank data field 41270). The estimated prevalence of individuals with ICD-10 coded diagnosis are 0.6% with SCZ or another psychotic disorder, 0.6% with BD, 5% with depression and less than 4% with anxiety disorder, as shown in Supplementary Table 1. The number of self-reported diagnoses is higher (Supplementary Table 2)^6^. However, the generalizability of these estimates is limited by the availability of self-report data from only a subset of the UK Biobank cohort. In addition, self-reported diagnoses should be considered with caution as they are less reliable than ICD-coded diagnoses. Therefore, any diagnostic classification arising from self-report should be regarded as probable, rather than a confirmed mental disorder^6^. For further information about self-reported diagnoses and symptoms in the UK Biobank, see Davis et al.^6^. In the loneliness GWAS, Day et al. performed a sensitivity analyses by repeating the GWAS without individuals with self-reported depression^5^. This sensitivity analysis did not result in any appreciable change in results, suggesting that depression did not confound the findings of loneliness loci^5^. Anxiety disorders did not seem to confound the findings either as most individuals with self-reported anxiety disorder were excluded when individuals with depression were removed from analysis, given the high comorbidity between these disorders^6^.

Further, we used data from GWASs on cardiovascular disease (CVD) risk factors, including body mass index (BMI) (n = 339 224)^7^, type 2 diabetes mellitus (T2D) (n = 159 208)^8^, total cholesterol (TC) (n = 188 578)^9^, high-density lipoprotein (HDL) cholesterol (n = 188 578)^9^, systolic blood pressure (SBP) (n = 200 000)^10^, diastolic blood pressure (DBP) (n = 200 000)^10^, along with coronary artery disease (CAD) (n =185 000)^11^. In addition, we used GWAS data on smoking (n > 200 000) for supplementary analysis^12^. We used a larger BMI GWAS (n = 795 640)^13^ including UK biobank data for MiXeR than for condFDR/conjFDR because MiXeR controls for overlapping samples and does, therefore, not require excluding overlapping participants (see below for description of MiXeR). The GWAS samples used for the present study are previously shown to be sufficiently powered to identify gene variants at genome-wide significant level. For further details of the inclusion criteria, genotyping and phenotype characteristics, see the original publications^1-3, 5, 7-13^.

**MiXeR**

We applied causal mixture models^14, 15^ to the GWAS summary statistics, using the MiXeR tool (<https://github.com/precimed/mixer>). For each SNP, $i$, univariate MiXeR models its additive genetic effect of allele substitution,$\beta_{i}$, as a point-normal mixture, $\beta_{i}=\left( 1-\pi_{1} \right)N\left( 0,0 \right)+\pi_{1}N(0, \sigma_{\beta}^{2})$, where $\pi_{1}$ represents the proportion of non-null SNPs (`polygenicity`) and $\sigma_{\beta}^{2}$ represents variance of effect sizes of non-null SNPs (`discoverability`). Then, for each SNP, $j$, MiXeR incorporates LD information and allele frequencies for M=9,997,231 SNPs extracted from 1000 Genomes Phase3 data by LD score regression software^16^, and estimate the expected probability distribution of the signed test statistic, $z_{j}=\delta_{j}+\epsilon_{j}=N\sum_{i} \sqrt{H_{i}}r_{ij}\beta_{i}+\epsilon_{j}$, where $N$ is sample size, $H_{i}$ indicates heterozygosity of i-th SNP, $r_{ij}$ indicates allelic correlation between i-th and j-th SNPs, and $\epsilon_{j}\sim N(0, \sigma_{0}^{2})$ is the residual variance. Further, the three parameters, $\pi_{1}, \sigma_{\beta}^{2}, \sigma_{0}^{2}$, are fitted by direct maximization of the likelihood function. The number of causal variants is estimated as $M\pi_{1}$, where M=9,997,231 gives the number of SNPs in the reference panel.

In the cross-trait analysis, MiXeR models additive genetic effects as a mixture of four components, representing null SNPs in both traits ($\pi_{0})$; SNPs with a specific effect on the first and on the second trait ($\pi_{1}$ and $\pi_{2}$, respectively); and SNPs with non-zero effect on both traits ($\pi_{12}$). In the last component, MiXeR models variance-covariance matrix as $\boldsymbol{\Sigma}_{\mathbf{12}}=\left[ \begin{matrix} \sigma_{1}^{2} & {\rho_{12}\sigma}_{1}\sigma_{2} \\ {\rho_{12}\sigma}_{1}\sigma_{2} & \sigma_{2}^{2} \end{matrix} \right]$ where $\rho_{12}$ indicates correlation of effect sizes within the shared component, and $\sigma_{1}^{2}$ and $\sigma_{2}^{2}$ correspond to the discoverability parameter estimated in the univariate analysis of the two traits. After fitting parameters of the model, the Dice coefficient of polygenic overlap is then calculated as $\frac{2\pi_{12}}{\pi_{1}+2\pi_{12}+\pi_{2}}$, and genetic correlation is calculated as $r_{g}=\frac{\rho_{12}\pi_{12}}{\sqrt{(\pi_{1}+\pi_{12})(\pi_{2}+\pi_{12})}}.$ Further information is available in^14^.

To filter situations with insufficiently powered GWAS summary statistics, we use Akaike information criterion ($AIC=2k-2\ln L$), where $k$ is the number of free parameters in the model, $L$ is the value of the likelihood function, and $n$ is the effective number of SNPs used in optimization procedure. We calculate the difference between *AIC* for the full bivariate model, $k=3$, and AIC for the reduced bivariate model, $k=2$, due to $\pi_{12}$ being constrained to smallest or largest possible ( $\pi_{12}^{min}=r_{g}\sqrt{\pi_{1}^{u} \pi_{2}^{u}}$ and $\pi_{12}^{max}=min(\pi_{1}^{u}, \pi_{2}^{u})$, respectively). A positive value of AIC indicates that GWAS summary statistics have enough information to distinguish the custom polygenic overlap, as shown on the MiXeR Venn diagrams, versus the constrained models with minimal ($\pi_{12}^{min}$) and maximum ($\pi_{12}^{max}$) polygenic overlap. MiXeR results are presented as a Venn diagram of shared and unique polygenic components across traits.

**Conditional False Discovery Rate**

The ‘enrichment’ seen in the conditional Q-Q plots can be directly interpreted in terms of true discovery rate (TDR = 1 – false discovery rate (FDR))^17^. More specifically, for a given p-value cutoff, the FDR is defined as

FDR(p) = π_0_F_0_(p) / F(p), [1]

where π_0_ is the proportion of null SNPs, F_0_ is the null cumulative distribution function (cdf), and F is the cdf of all SNPs, both null and non-null^18^. Here, we assume the SNP *p* values are a priori independent and identically distributed. Under the null hypothesis, F_0_ is the cdf of the uniform distribution on the unit interval [0,1], so that Eq. [1] reduces to

FDR(p) = π_0_p / F(p), [2]

The cdf F can be estimated by the empirical cdf q = N_p_ / Ν, where N_p_ is the number of SNPs with p-values < p, and N is the total number of SNPs. Replacing F by q in Eq. [2], we get

Estimated FDR(p) = π_0_p / q, [3]

which is biased upwards as an estimate of the FDR^19^. Replacing π_0_ in Equation [3] with unity gives an estimated FDR that is further biased upward;

q* = p / q, [4]

If π_0_ is close to one, which is probably true for most GWASs, the increase in bias from Eq. [3] is minimal. Therefore, the quantity 1 – p/q, is biased downward and thus a conservative estimate of the TDR. Referring to the Q-Q plots, we see that q* is equivalent to the nominal p-value divided by the empirical quantile, as defined previously. We can thus read the FDR estimate directly off the Q-Q plot as

-log_10_(q*) = log_10_(q) – log_10_(p), [5]

demonstrating that the estimated FDR is directly related to the horizontal shift of the curves in the Q-Q plots from the expected line x = y, i.e. a larger shift corresponds to a smaller FDR.

**Conditional Q-Q plots**

Q-Q plots compare a nominal probability distribution against an empirical distribution. In the presence of all null relationships, nominal p-values form a straight line on a Q-Q plot when plotted against the empirical distribution. For SCZ, BD, MD, loneliness and CVD risk factor SNPs and for each categorical subset (strata), -log_10_ nominal p-values were plotted against -log_10_ empirical p-values (conditional Q-Q plots). Leftward deflections of the observed distribution from the projected null line illustrate increased tail probabilities in the distribution of test statistics (z-scores) and consequently an over-abundance of low p-values compared to that expected by chance, also called ‘enrichment’.

Under large-scale testing paradigms, such as GWAS, we can calculate quantitative estimates of likely true associations from the distributions of summary statistics^18, 20^. Conditional Q-Q plots of nominal p-values from GWAS summary statistics visualizes this enrichment of statistical association relative to that expected under the global null hypothesis. The usual Q-Q curve has the nominal p value, denoted by "p", as the y-ordinate and the corresponding value of the empirical cdf, denoted by "q", as the x-ordinate. Under the global null hypothesis the theoretical distribution is uniform on the interval [0,1]. As is common in GWAS, we instead plot -log_10_ p against -log_10_ q to emphasize tail probabilities of the theoretical and empirical distributions. Therefore, genetic enrichment is illustrated with a leftward shift in the Q-Q curve, corresponding to a larger fraction of SNPs with nominal -log_10_ p-value greater than or equal to a given threshold. Conditional Q-Q plots are constructed by creating subsets of SNPs based on levels of an auxiliary measure for each SNP, and computing Q-Q plots separately for each level. If SNP enrichment is captured by variation in the auxiliary measure, this is expressed as successive leftward deflections in a conditional Q-Q plot as levels of the auxiliary measure increase. We constructed conditional Q-Q plots of empirical quantiles of nominal -log_10_ values for SNP association for all SNPs, and for subsets (strata) of SNPs determined by the nominal p-values of their association with the conditional phenotypes, and vice versa. In particular, we computed the empirical cumulative distribution (cdf) of nominal p-values for a given phenotype for all SNPs and for SNPs with significance levels below the indicated cut-offs for the conditional phenotypes (-log_10_(p) ≥ 1, -log_10_(p) ≥ 2, -log_10_(p) ≥ 3 corresponding to p < 0.1, p < 0.01, p < 0.001 respectively). The nominal p-values (–log_10_(p)) are plotted on the y-axis, and the empirical quantiles (–log_10_(q), where q=1-cdf(p)) are plotted on the x-axis. To assess for polygenic effects below the standard GWAS significance threshold, we focused the conditional Q-Q plots on SNPs with nominal –log_10_(p) < 7.3 (corresponding to p > 5x10^-8^). We controlled for spurious enrichment by calculating all conditional Q-Q plots after random pruning averaged over 500 iterations. At each iteration, one SNP in every LD block (defined by an r^2^ >0.1) was randomly selected and the empirical cdfs were computed using the corresponding p-values.

**Detection of SNPs using conditional and conjunctional FDR**

The FDR can be interpreted as the probability that a SNP is null given that its p-value is as small as or smaller than its observed p-value. The conditional FDR (condFDR) is an extension of the standard FDR, which incorporates information from GWAS summary statistics of a second phenotype to adjust its significance level. The condFDR is defined as the probability that a SNP is null in the first phenotype given that the p-values in the first and second phenotypes are as small as or smaller than the observed ones. It is important to note that ranking SNPs by the standard FDR or by p-values gives the same ordering of SNPs. In contrast, ranking SNPs by condFDR will reorder SNPs when the primary and secondary phenotypes are genetically related.

To identify SNPs that are associated with *both* phenotypes, we used conjunctional FDR (conjFDR)^21, 22^, employing an overall FDR threshold of 0.05 according to the standard FDR approach^4^. The conjunctional FDR (conjFDR) is defined as the posterior probability that a SNP is null for either phenotype or both simultaneously, given that its p-values for association with both phenotypes are as small as or smaller than the observed p-values^21, 23-26^. A conservative estimate of the conjFDR is obtained by the maximum condFDR for a given SNP after repeating the condFDR procedure for both traits and inverting their roles^27^. Given that complex correlations in regions with intricate LD can bias FDR estimation^28^, we excluded SNPs in the extended major histocompatibility complex and chromosome 8p23.1 (genome build 19 locations 25119106–33854733 and 7242715–12483982, respectively) and SNPs in LD (r^2^>0.1) with such SNPs before fitting the FDR models. To investigate loci shared between three phenotypes, including SMDs, loneliness and BMI, we used trio conjFDR. Trio conjFDR value of an SNP is defined as the maximum of three pairwise conjFDR values of the SNP. For example, trio conjFDR_{SCZ & BMI& loneliness}c_ = max(conjFDR_{SCZ & BMI}_, conjFDR_{SCZ & loneliness}_, conjFDR_{BMI & loneliness}_). Effect size (z-scores) of SNPs were obtained from the original summary statistics (see original publications for how they were calculated^1-3, 5, 7-13^). P-values were corrected for inflation using a genomic inflation control procedure^21^.

**Genomic loci definition**

We defined independent genomic loci using the FUMA, an online tool for functional mapping of genetic variants (<http://fuma.ctglab.nl/>)^29^. Summary statistics from the GWASs on SMDs, loneliness and CVD risk factors were used as input for FUMA. First, *independent* *significant SNPs* were identified as SNPs with condFDR < 0.01 and independent from each other at LD r^2^ < 0.6. Secondly, *lead SNPs* were identified by retaining those independent significant SNPs that were independent from each other at r^2^ < 0.1. Next, *distinct genomic loci* were identified by merging physically overlapping lead SNPs (LD blocks < 250 kb apart). Borders of the genomic loci were determined by identifying all SNPs in LD (r^2^ ≧ 0.6) with one of the independent significant SNPs in the locus. The region containing all of these *candidate SNPs* was regarded as a single independent genomic locus. All LD information was calculated from the 1000 Genomes Project reference panel^30^.

**Genetic correlation**

We estimated the genetic correlation using MiXeR^14^ and LD score regression^31^, procedures that control for overlapping samples without requiring individual genotype data. LD score regression was estimated using the Python-based package available at https://github.com/bulik/ldsc. The procedure is described in the documentation of the package (https://github.com/bulik/ldsc/wiki/Heritability-and-Genetic-Correlation).

**Functional annotation**

We used FUMA^29^, an online annotation platform (http://fuma.ctglab.nl/) to functionally annotate all candidate SNPs in the genomic loci with a condFDR or conjFDR value<0.10 having an r^2^≧0.6 with one of the independent significant SNPs. SNPs were annotated with Combined Annotation Dependent Depletion (CADD) scores^32^, RegulomeDB^33^ scores, and chromatin states^34, 35^ (see below). We conducted gene-set analysis to evaluate whether the genes mapped to the shared loci were overrepresented via FUMA^29^. We used Bonferroni-adjusted p-value threshold of 0.05 to correct for multiple comparisons.

The CADD score is a deleterious score of variants computed by integrating 63 functional annotations^32^. The higher the score, the more deleterious. A CADD score above 12.37 is the threshold to be potentially pathogenic^32^. The RegulomeDB score is a categorical score to guide interpretation of regulatory variants^33^. It is based on information from eQTLs and chromatin marks, ranging from 1a to 7 with lower scores indicating a higher likelihood of having a regulatory function. Scores are as follows: 1a=eQTL + Transcription Factor (TF) binding + matched TF motif + matched DNase Footprint + DNase peak; 1b=eQTL + TF binding + any motif + DNase Footprint + DNase peak; 1c=eQTL + TF binding + matched TF motif + DNase peak; 1d=eQTL + TF binding + any motif + DNase peak; 1e=eQTL + TF binding + matched TF motif; 1f=eQTL + TF binding / DNase peak; 2a=TF binding + matched TF motif + matched DNase Footprint + DNase peak; 2b=TF binding + any motif + DNase Footprint + DNase peak; 2c=TF binding + matched TF motif + DNase peak; 3a=TF binding + any motif + DNase peak; 3b=TF binding + matched TF motif; 4=TF binding + DNase peak; 5=TF binding or DNase peak; 6=other; 7=Not available^33^.

The chromatin state represents the accessibility of genomic regions (every 200bp) with 15 categorical states predicted by a hidden Markov model based on 5 chromatin marks for 127 epigenomes in the Roadmap Epigenomics Project^35^. A lower state indicates increased accessibility, with states 1-7 referring to open chromatin states. We annotated the minimum chromatin state across tissues to SNPs. The 15-core chromatin states as suggested by Roadmap are as follows: 1=Active Transcription Start Site (TSS); 2=Flanking Active TSS; 3=Transcription at gene 5’ and 3’; 4=Strong transcription; 5= Weak Transcription; 6=Genic enhancers; 7=Enhancers; 8=Zinc finger genes & repeats; 9=Heterochromatic; 10=Bivalent/Poised TSS; 11=Flanking Bivalent/Poised TSS/Enh; 12=Bivalent Enhancer; 13=Repressed PolyComb; 14=Weak Repressed PolyComb; 15=Quiescent/Low.

We also used FUMA to link candidate and lead SNPs to genes using either of three gene-mapping strategies: 1) positional mapping to link SNPs to genes based on their physical proximity (i.e., within a 10kb window), 2) expression quantitative trait locus (eQTL) mapping to match cis-eQTL SNPs to genes whose expression is associated with allelic variation at the SNP level, and 3) chromatin interaction mapping to link SNPs to genes based on three-dimensional DNA–DNA interactions between each SNP’s genomic region and nearby or distant genes, as used in a recent GWAS from our group^36^. We considered eleven eQTL databases in FUMA which include eQTL information from several human tissue types including multiple brain regions (http://fuma.ctglab.nl/tutorial#eQTLs). The eQTL analyses were corrected for multiple comparisons using an FDR threshold of 0.05. FUMA includes Hi-C data of over 21 tissue/cell types including human brain tissue (https://fuma.ctglab.nl/tutorial#chromatin-interactions). We used an FDR of 1 x 10^-6^ to define significant chromatin interactions, in line with recommendations^37^. Analyses were corrected for multiple comparisons.

**Image processing software**

Matplotlib Python library (https://matplotlib.org/) and Matlab.

**SUPPLEMENTARY RESULTS**

**MiXeR results**

The MiXeR model provides adequate fit to the GWAS data of loneliness and SMDs, as indicated by AIC in Supplementary Table 3, conditional Q-Q plots and negative log-likelihood (Supplementary Figures 3-5), while the results of loneliness vs MD analysis are more uncertain, suggesting that a larger MD GWAS is needed to obtain more certain MiXeR estimates. In particular, the negative values of AIC indicate that GWAS summary statistics do not have enough power to distinguish the estimated polygenic overlap, as shown on the MiXeR Venn diagrams, versus the constrained models with minimal ($\pi_{12}^{\min}$) and maximum ($\pi_{12}^{\max}$) polygenic overlap (Supplementary Table 3). Nevertheless, the Venn diagram suggests that loneliness and MD share genetic architecture (Figure 1a), although the amount of shared genetic variants remains uncertain, as indicated by AIC. Further, the MiXeR results for BMI should be interpreted with some caution because the MiXeR model was less accurate in predicting the empirical data (see conditional Q–Q plot in Supplementary Figure 8) than the MiXeR model for loneliness and SMDs (Supplementary Figure 3-5). Further information about the quality of the MiXeR model for loneliness and BMI, see Supplementary Figure 8 and AIC in Supplementary Table 3.

**Gene-mapping results**

We performed gene-mapping of lead and candidate SNPs, which provided consistent results, implicating brain-expressed genes. Among lead SNPs shared between loneliness and MD (67), positional mapping aligned the SNPs to 44 genes, cis-eQTL mapping implicated 20 genes, and chromatin interaction mapping implicated 5 genes (Supplementary Table 17). Of SNPs shared with SCZ (54), positional mapping aligned the SNPs to 34 genes, cis-eQTL mapping implicated 20 genes, and chromatin interaction mapping implicated 7 genes (Supplementary Table 18). Among SNPs shared with BD (28), positional mapping linked the SNPs to 14 genes, cis-eQTL mapping indicated 13 genes, and chromatin interaction mapping implicated 2 genes (Supplementary Table 19). Taken together, 69.8% (104/149) of the SNPs shared between loneliness and SMDs were mapped to genes when considering all gene-mapping strategies, of which approximately 40.3 % of them mapped to brain-expressed genes (60/149) based on eQTL and chromatin interaction mapping (Supplementary Tables 17-19). Among SNPs shared between loneliness and BMI (36), positional mapping aligned the SNPs to 25 genes, cis-eQTL mapping implicated 14 genes, and chromatin interaction mapping implicated 1 gene (Supplementary Table 20). Of the SNPs shared between loneliness and the remaining CVD risk factors and CAD, all SNPs were mapped to genes when considering all three gene-mapping strategies (Supplementary Tables 21-26). The majority of these SNPs were mapped to genes with eQTL and chromatin interaction mapping, indicating brain-expressed genes (TC: 6/6; HDL-C: 3/5, SBP: 5/9; DBP: 3/4; CAD: 8/12; and T2D: 1/1).

Among lead SNPs shared between loneliness, BMI and MD, positional mapping aligned the SNPs to 2 genes, while no SNPs were gene-mapped based on the other two strategies (Supplementary Table 27). Among SNPs shared between loneliness, BMI and SCZ, positional mapping linked the SNPs to 3 genes, 2 of which were mapped using eQTL and chromatin interaction mapping (Supplementary Table 28). No SNPs shared between loneliness, BMI and BD were mapped to genes (Supplementary Table 29).

Among candidate SNPs shared between loneliness and MD (4256), positional mapping aligned the SNPs to 2895 genes, cis-eQTL mapping implicated 1737 genes, and chromatin interaction mapping implicated 124 genes (Supplementary Table 30). Of candidate SNPs shared with SCZ (4021) positional mapping aligned the SNPs to 2273 genes, cis-eQTL mapping implicated 1851 genes, and chromatin interaction mapping implicated 201 genes (Supplementary Table 31). Among the candidate SNPs shared with BD (1349), positional mapping aligned the SNPs to 532 genes, cis-eQTL mapping implicated 489 genes, and chromatin interaction mapping implicated 25 genes (Supplementary Table 32). Taken together, 69.8% (6716/9626) of the SNPs shared between loneliness and SMDs were mapped to genes when considering all gene-mapping strategies, of which approximately 42.8 % of them mapped to genes (4219/9626) based on eQTL and chromatin interaction mapping (Supplementary Tables 30-32). Further, among the candidate SNPs shared with BMI (1037), positional mapping aligned the SNPs to 732 genes, cis-eQTL mapping implicated 450 genes, and chromatin interaction mapping implicated 46 genes (Supplementary Table 33). Of the SNPs shared between loneliness and the remaining CVD risk factors and CAD, all SNPs were mapped to genes (expect for 22% of CAD SNP shared with loneliness) when considering all three gene-mapping strategies (Supplementary Tables 34-39). The majority of these SNPs were mapped to genes with eQTL and chromatin interaction mapping (TC: 187/190; HDL-C: 181/194, SBP: 503/519: DBP: 120/122; CAD: 700/996; and T2D: 8/8).

**Gene-set analysis results**

For gene-set analyses, we focused on the genes mapped to the loci shared between loneliness and SMDs and BMI, as these phenotypes showed most genetic overlap in the above results. Gene-set analyses for genes mapped to loci shared between loneliness and SMDs discovered several biological and cellular processes, including “chromatin assembly”, “nucleosome organization”, “negative regulation of biosynthetic process” and “DNA packaging complex” (Supplementary Tables 40-42). Other significant processes involved “immune system development” and neural processes (e.g. “synapse”, “postsynapse” and “dendritic tree”) (Supplementary Tables 40-42). Further, genes mapped to loci shared between loneliness and BMI, were significantly associated with four biological processes, the most strongly associated being “positive regulation of RNA biosynthetic process” (Supplementary Table 43), suggesting metabolic processes.

**SUPPLEMENTARY FIGURES**


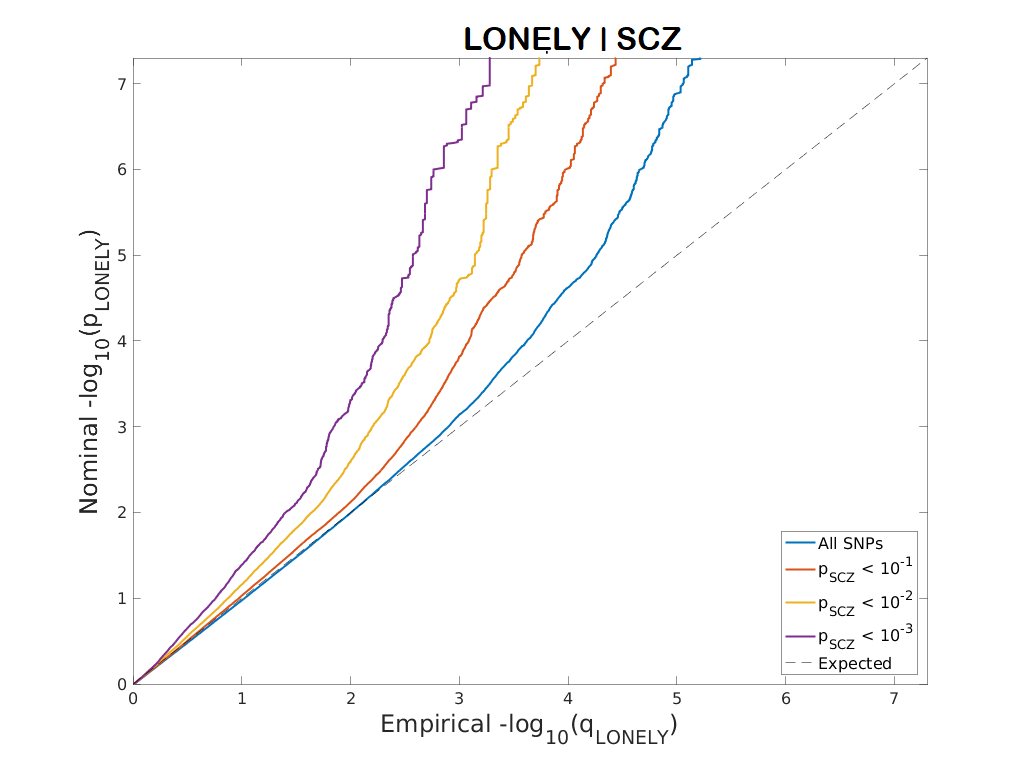

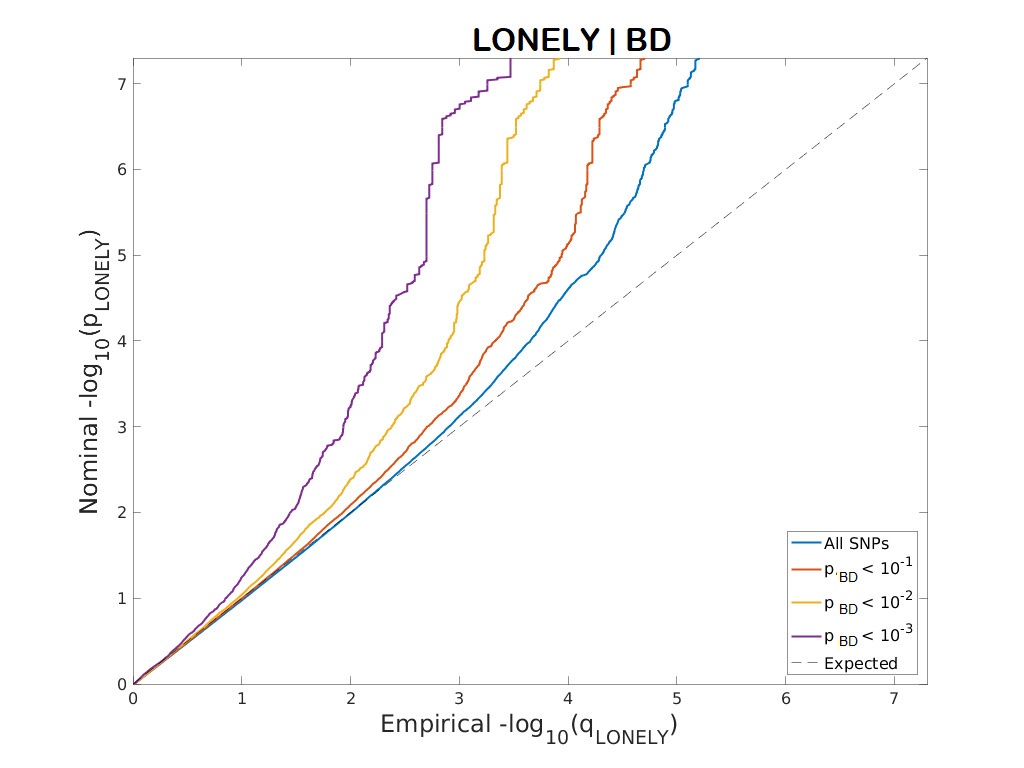

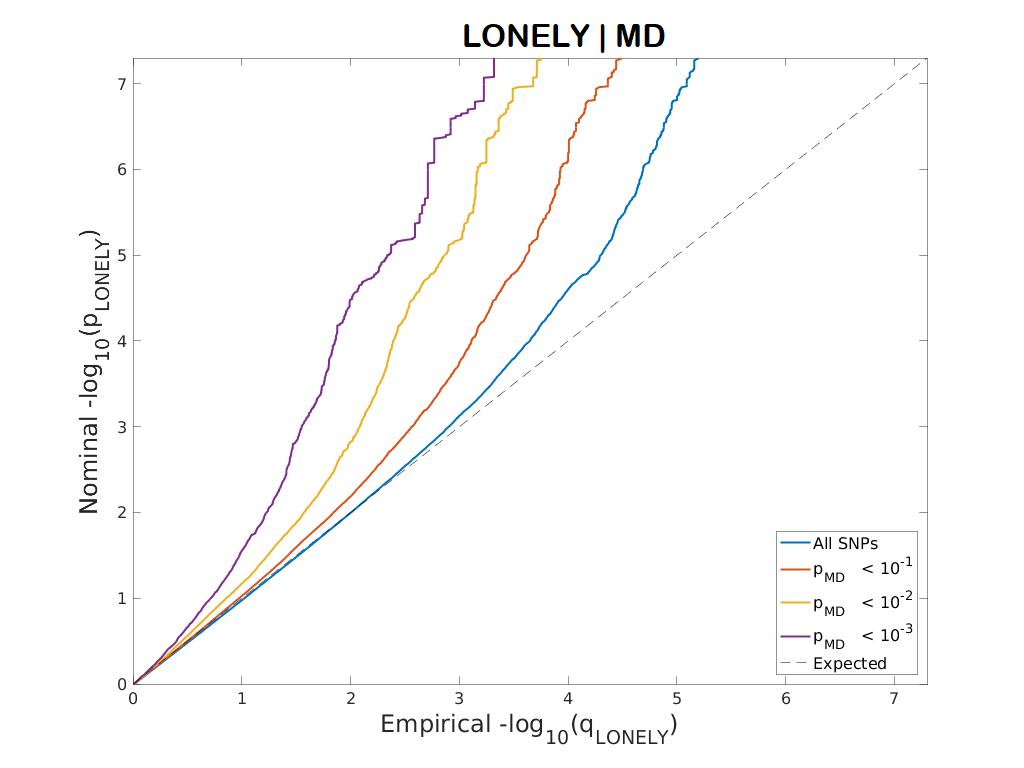


**Supplementary Figure 1.** Polygenic overlap between loneliness and MD, SCZ and BD. Conditional Q-Q plots of nominal versus empirical −log10p values (corrected for inflation) in loneliness below the standard GWAS threshold of p < 5 × 10−8 as a function of significance of association with MD, SCZ and BD at the level of p < 0.1, p < 0.01, p < 0.001, respectively. The blue lines indicate all SNPs. The dashed lines indicate the null hypothesis. Abbreviations: MD, major depression; SCZ, schizophrenia; BD, bipolar disorder. The conditional Q-Q plots build on the condFDR method.


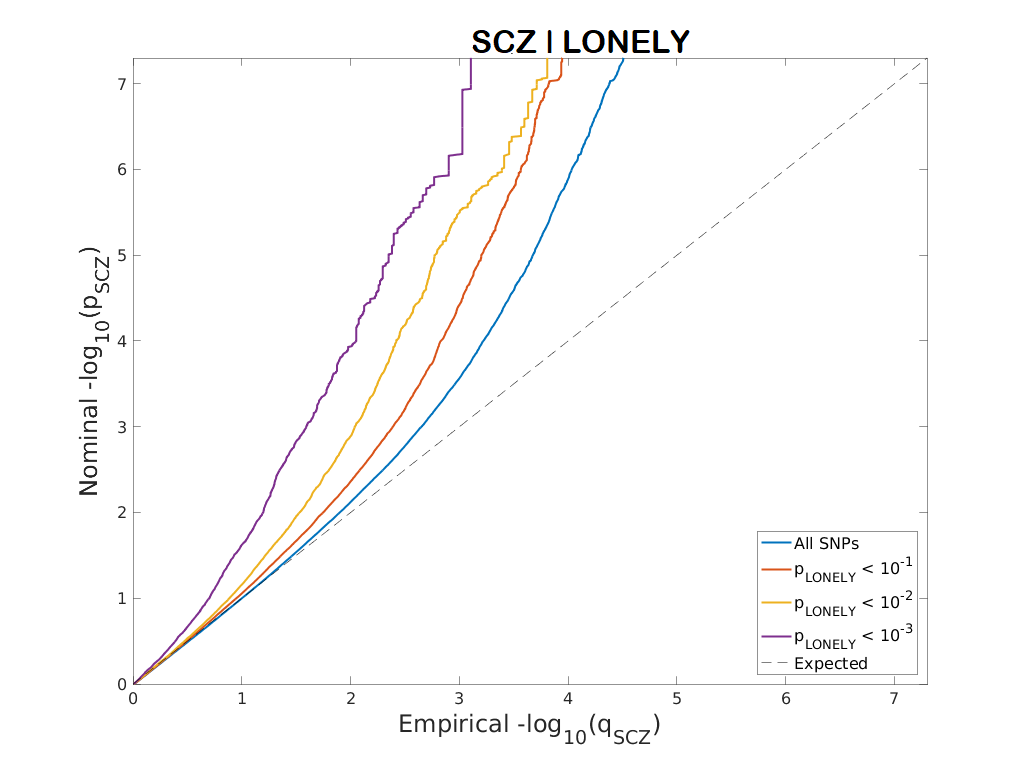

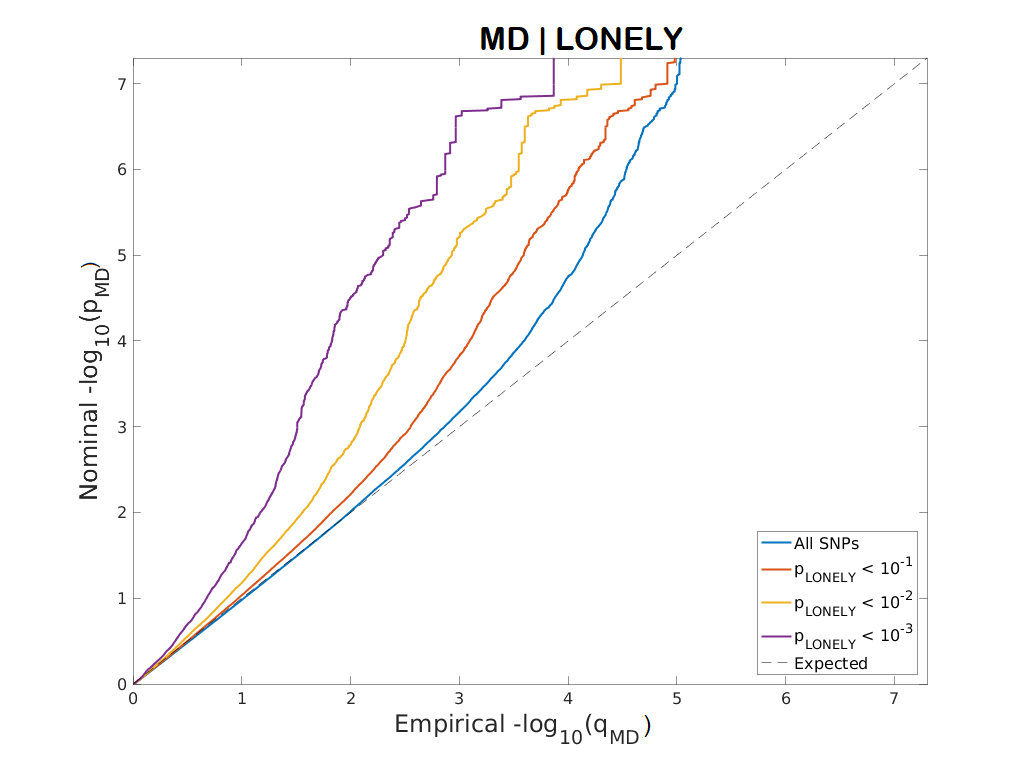

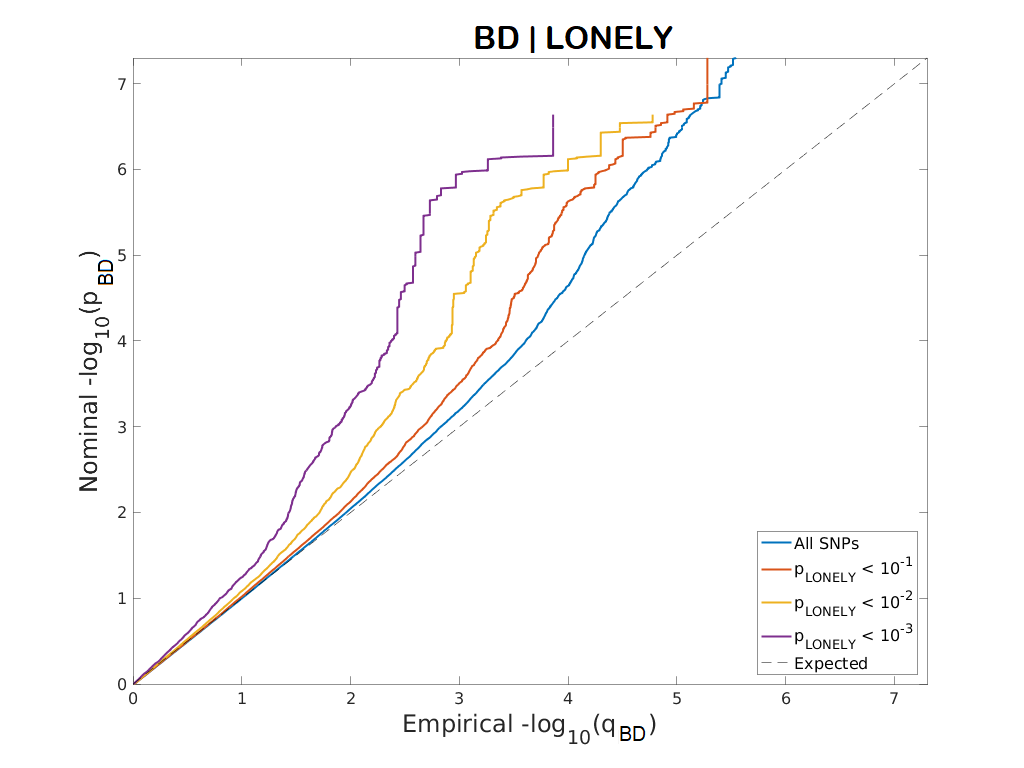


**Supplementary Figure 2.** Polygenic overlap between loneliness and MD, SCZ and BD. Conditional Q-Q plots of nominal versus empirical −log_10_ p-values (corrected for inflation) in MD, SCZ and BD below the standard GWAS threshold of p < 5×10^−8^ as a function of significance of association with loneliness, at the level of p < 0.1, p < 0.01, p < 0.001, respectively. The blue lines indicate all SNPs. The dashed lines indicate the null hypothesis. Abbreviations: MD, major depression; SCZ, schizophrenia; BD, bipolar disorder. The conditional Q-Q plots build on the condFDR method.


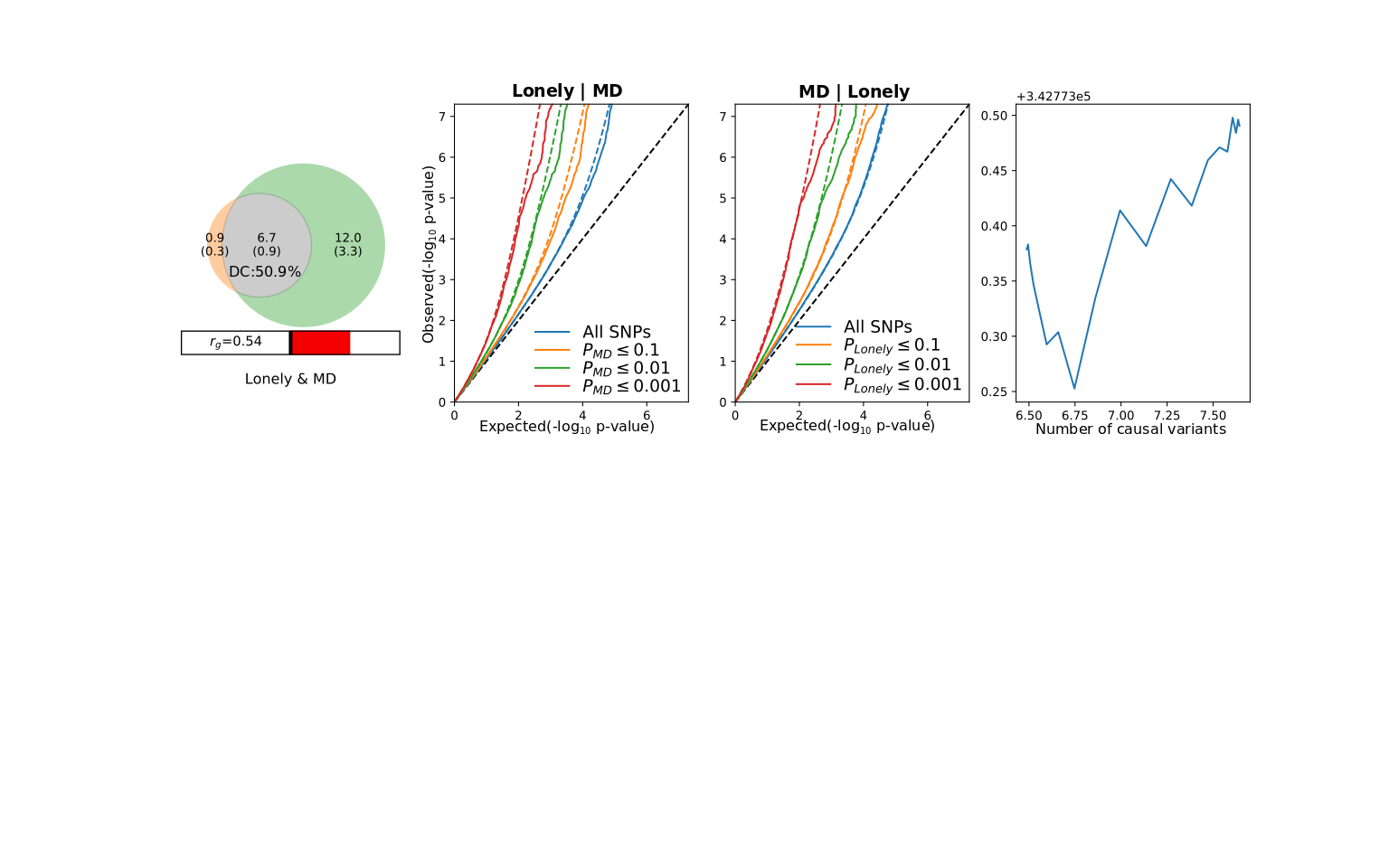


**Supplementary Figure 3**. Venn diagram, conditional Q-Q plots, and negative log-likelihood plot, respectively. *Venn diagram* of unique and shared polygenic components at the causal level, showing polygenic overlap (gray) between loneliness (orange) and major depression (MD) (green). The numbers in the Venn diagram indicate the estimated quantity of causal variants (in thousands) per component, explaining 90% of SNP heritability in each phenotype, followed by standard error. The size of the circles reflects the degree of polygenicity. The Dice coefficient (DC) in the Venn diagram indicates the percentage of shared causal variants between the two phenotypes. *Conditional Q–Q plots* of observed versus expected −log_10_ *p*-values in the primary trait as a function of significance of association with a secondary trait at the level of p < 0.1, p < 0.01, p < 0.001. Blue line indicates all SNPs. Dotted lines in blue, orange, green, and red indicate model predictions for each stratum. Black dotted line is the expected Q–Q plot under null hypothesis. *Negative log-likelihood plot*: minus log-likelihood calculated for the bivariate model as a function of 𝜋 parameter. The remaining parameters of the model were constrained to their fitted values. Figures generated from MiXeR.


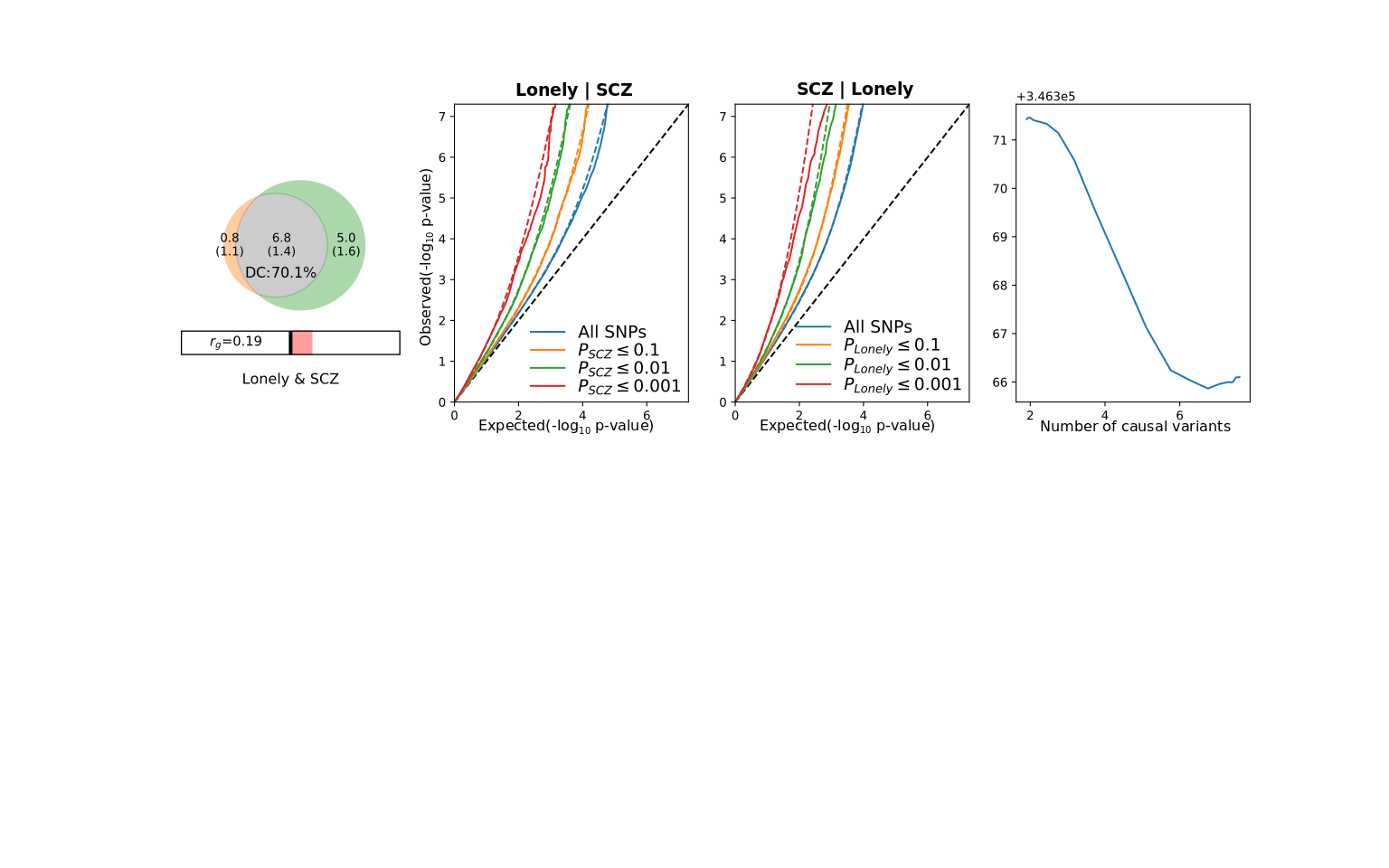


**Supplementary Figure 4**. Venn diagram, conditional Q-Q plots, and negative log-likelihood plot, respectively. Venn diagram of unique and shared polygenic components at the causal level, showing polygenic overlap (gray) between loneliness (orange) and schizophrenia (SCZ) (green). The numbers in the Venn diagram indicate the estimated quantity of causal variants (in thousands) per component, explaining 90% of SNP heritability in each phenotype, followed by standard error, and Dice coefficient (DC) indicates the percentage of shared causal variants between the two phenotypes. Appearance of the Q-Q plot and negative log-likelihood plot are described below the previous figure (Supplementary Figure 3). Figures generated from MiXeR.

**Supplementary Figure 5**. Venn diagram, conditional Q-Q plots, and negative log-likelihood plot, respectively. Venn diagram of unique and shared polygenic components at the causal level, showing polygenic overlap (gray) between loneliness (orange) and bipolar disorder (BD) (green). The numbers indicate the estimated quantity of causal variants (in thousands) per component, explaining 90% of SNP heritability in each phenotype, followed by standard error, and Dice coefficient (DC) indicates the percentage of shared causal variants between the two phenotypes. Appearance of the Q-Q plot and negative log-likelihood plot are described below Supplementary Figure 3. Figures generated from MiXeR.


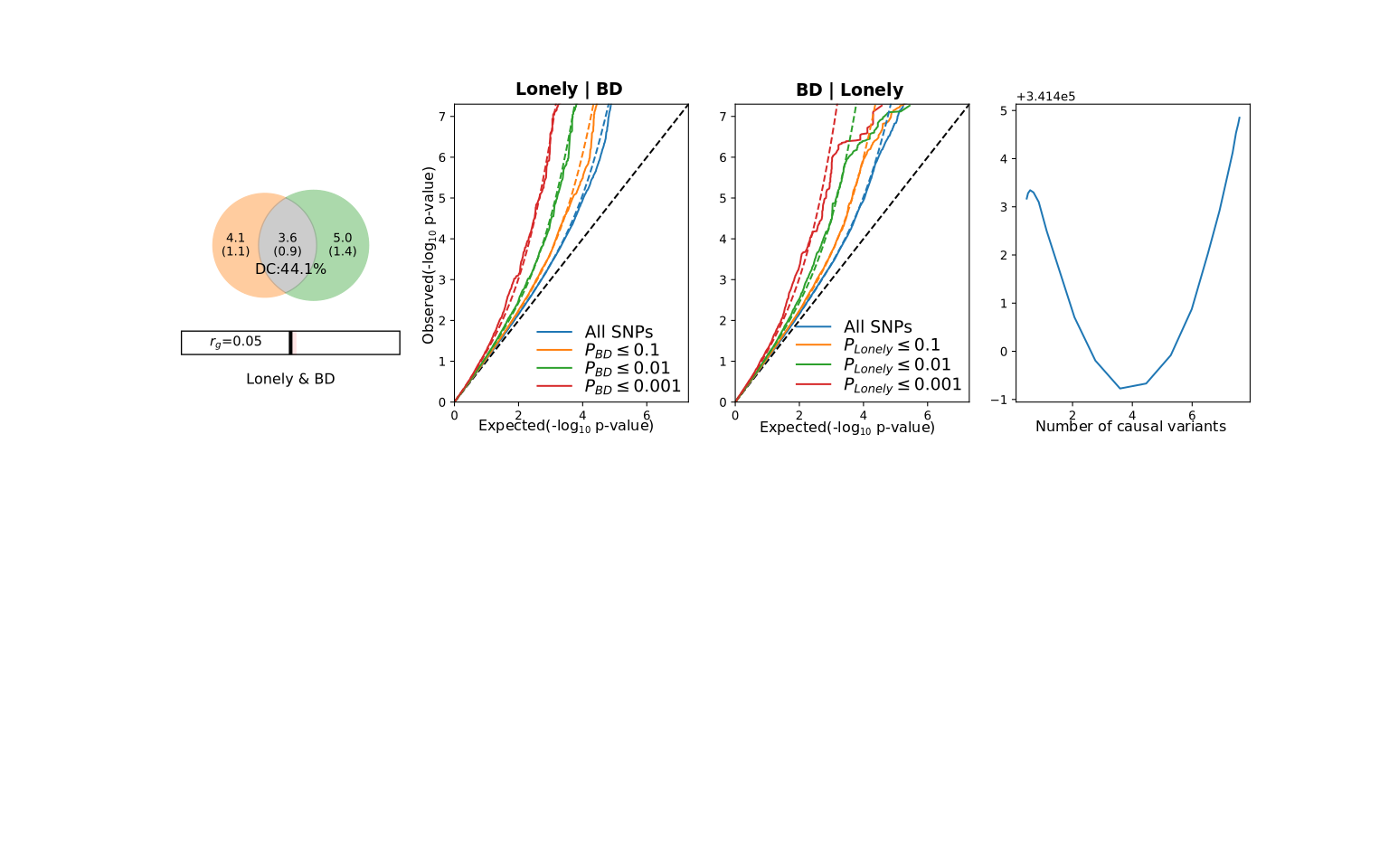

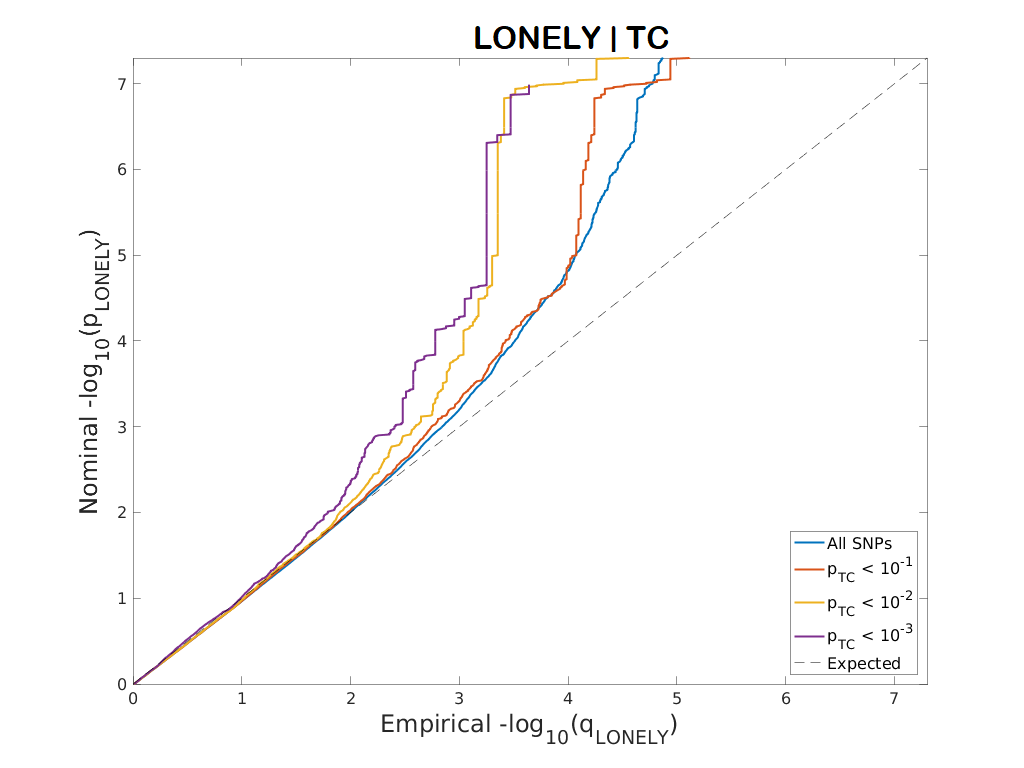

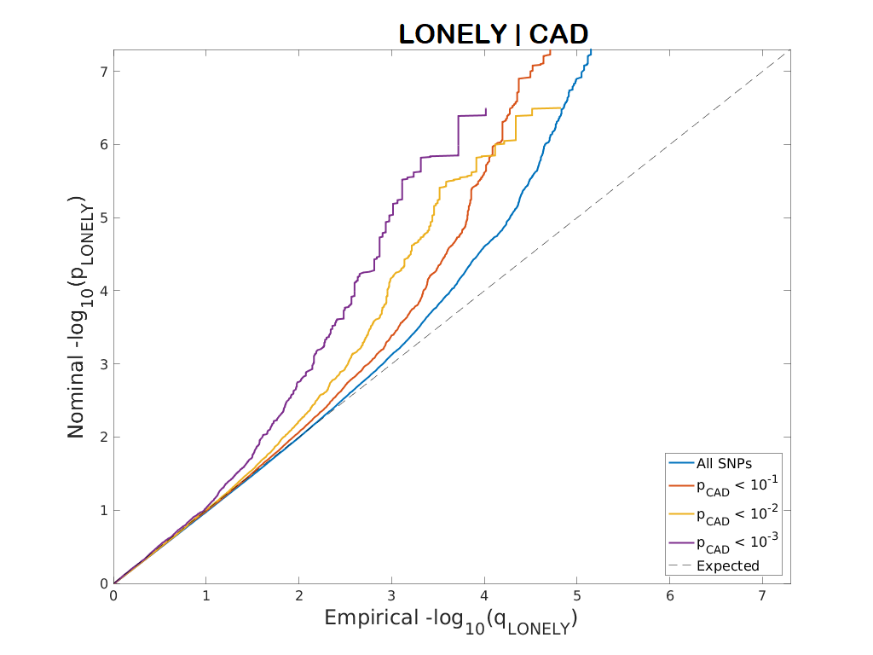

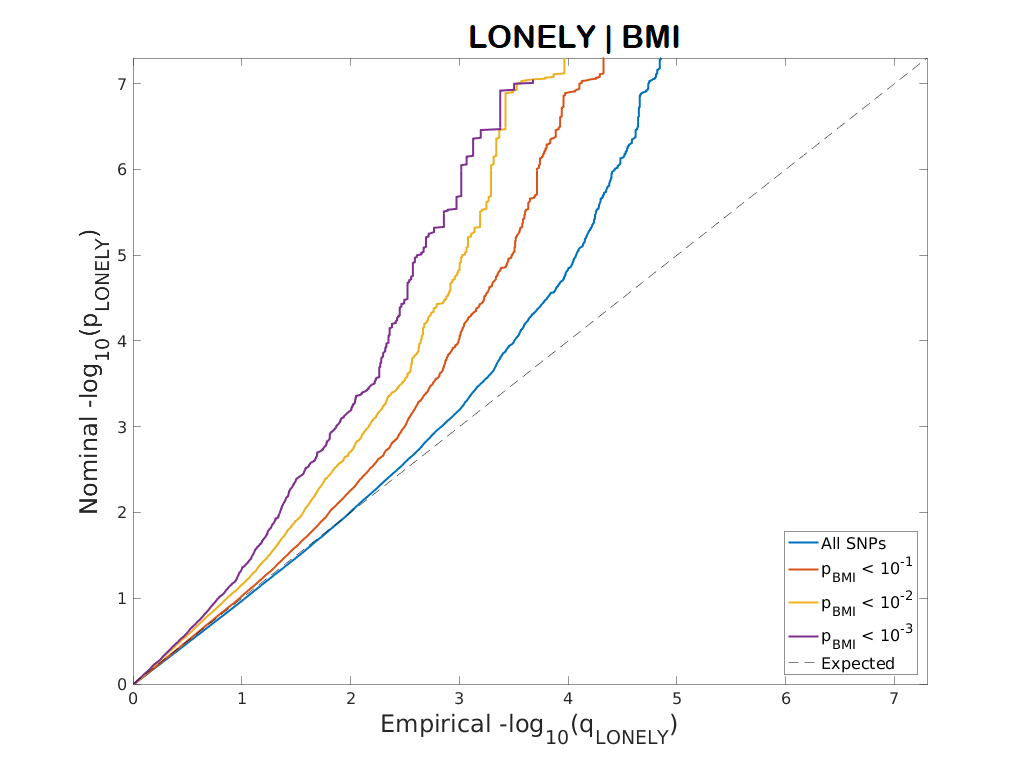

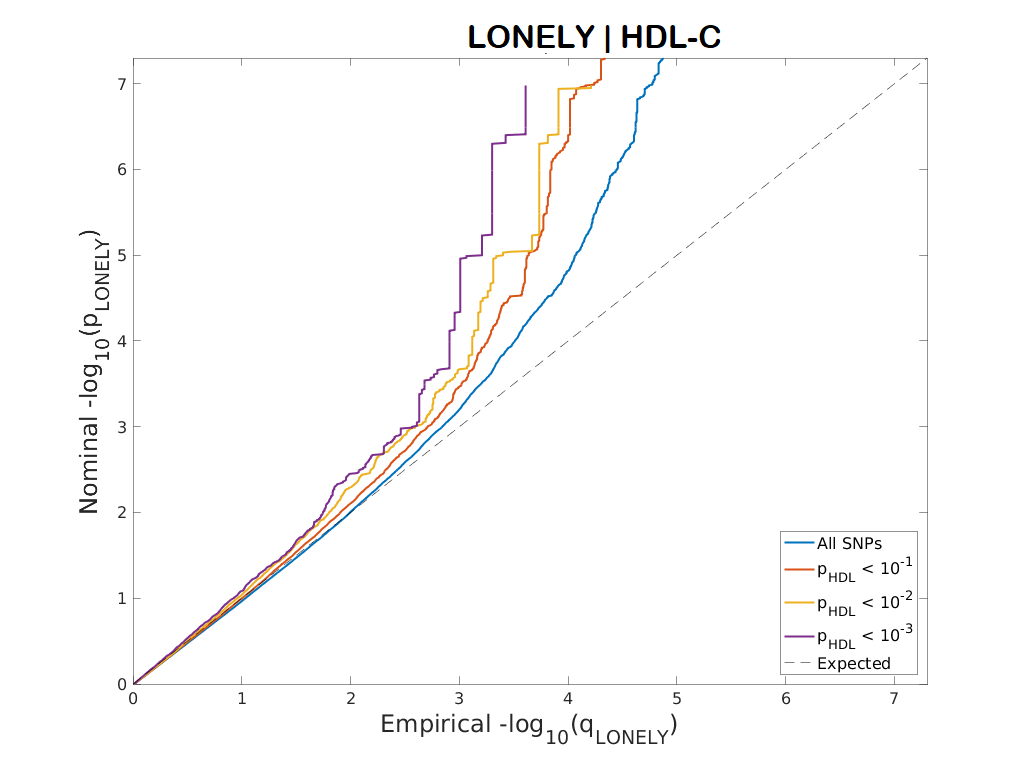

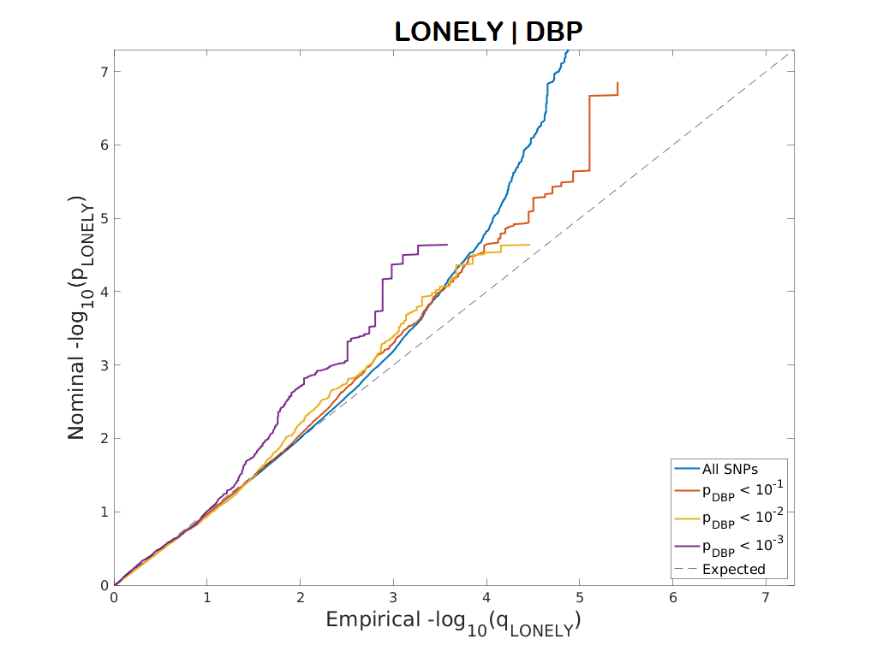

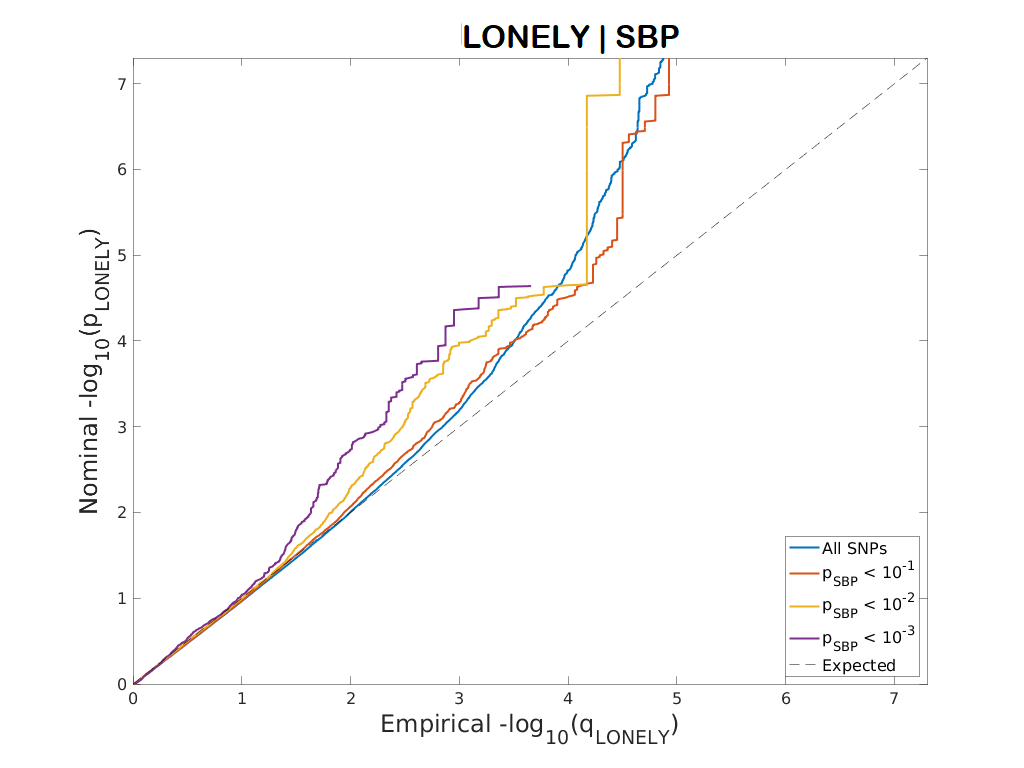


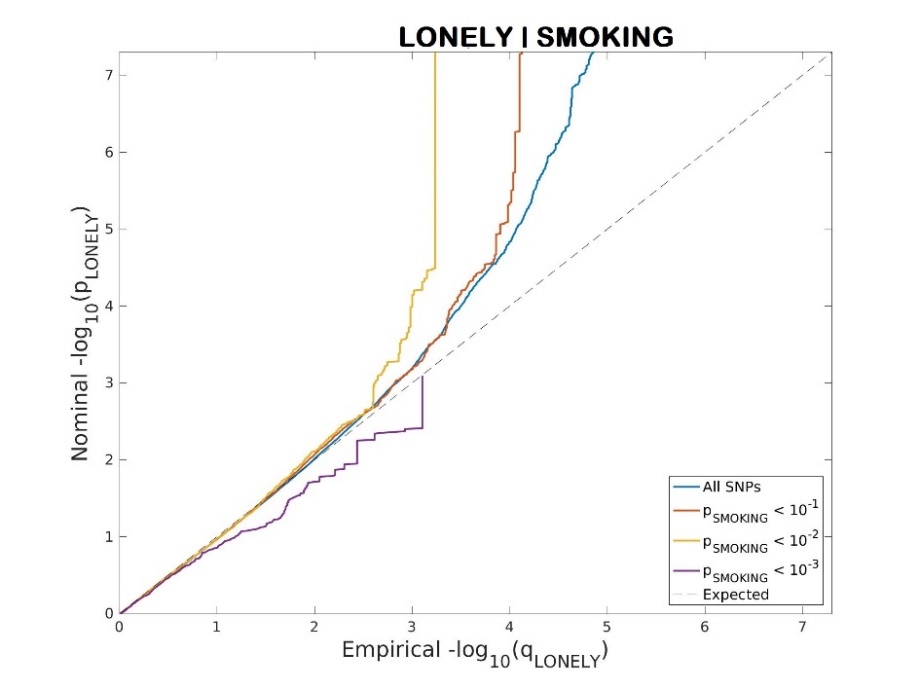

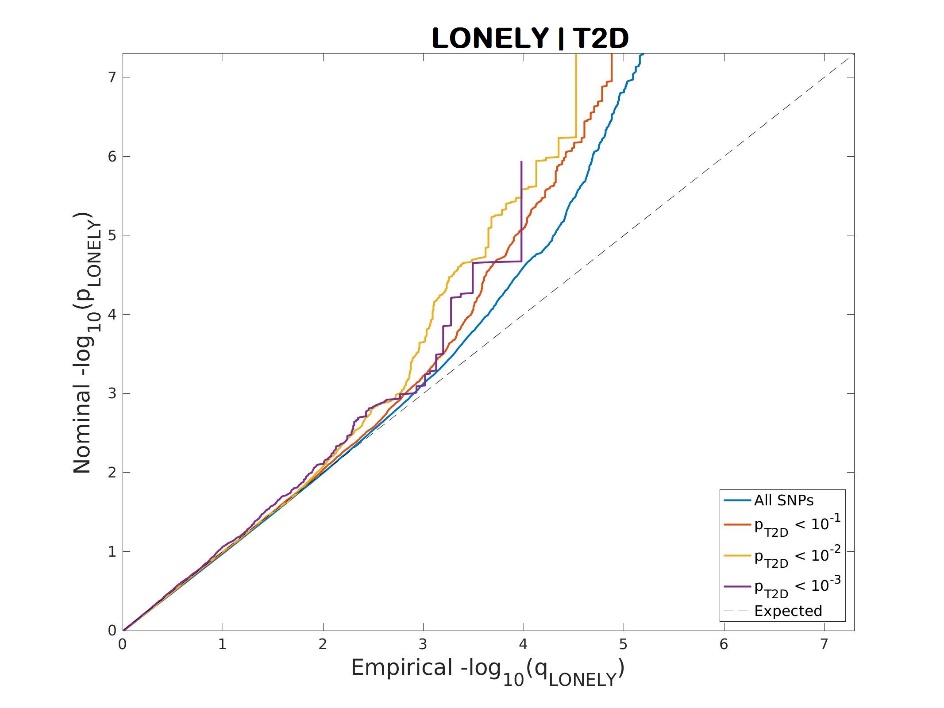


**Supplementary Figure 6.** Polygenic overlap between loneliness and CVD risk factors. Conditional Q-Q plots of nominal versus empirical −log_10_ p-values (corrected for inflation) in loneliness below the standard GWAS threshold of p < 5×10^−8^ as a function of significance of association with CVD risk factors at the level of p < 0.1, p < 0.01, p < 0.001, respectively. The blue lines indicate all SNPs. The dashed lines indicate the null hypothesis. Abbreviations: CVD, cardiovascular disease; BMI, body mass index; CAD, coronary heart disease; HDL-C, high-density lipoprotein cholesterol; TC, total cholesterol; SBP, systolic blood pressure; DBP, diastolic blood pressure; T2D, type 2 diabetes mellitus. The conditional Q-Q plots build on the condFDR method.


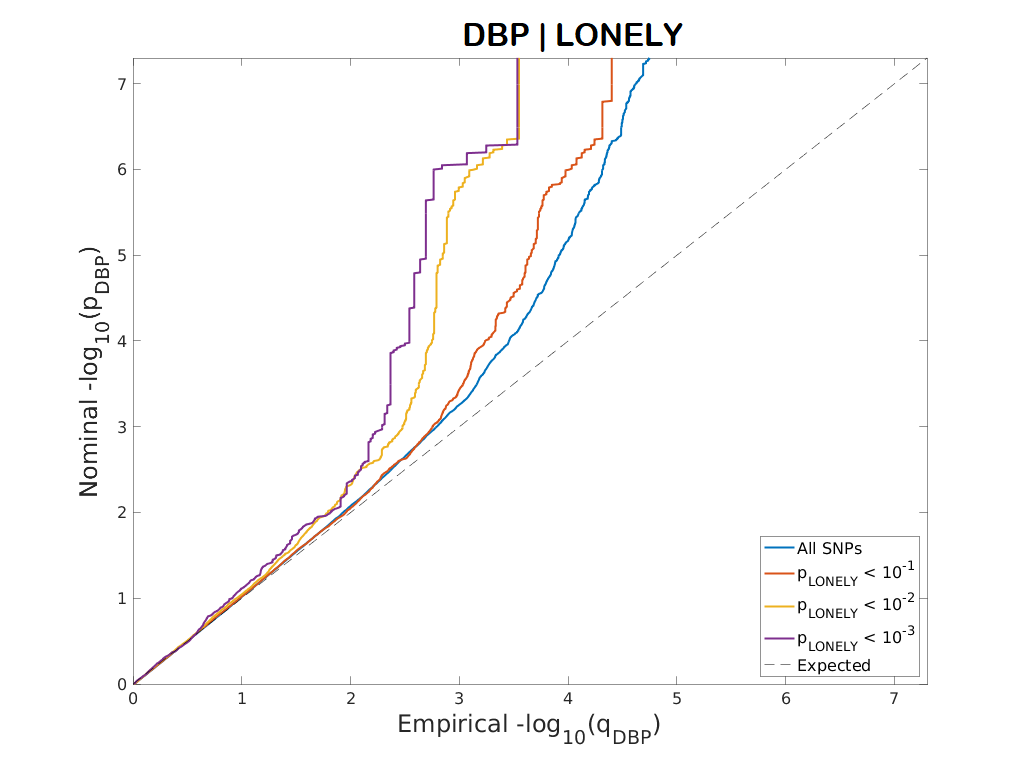

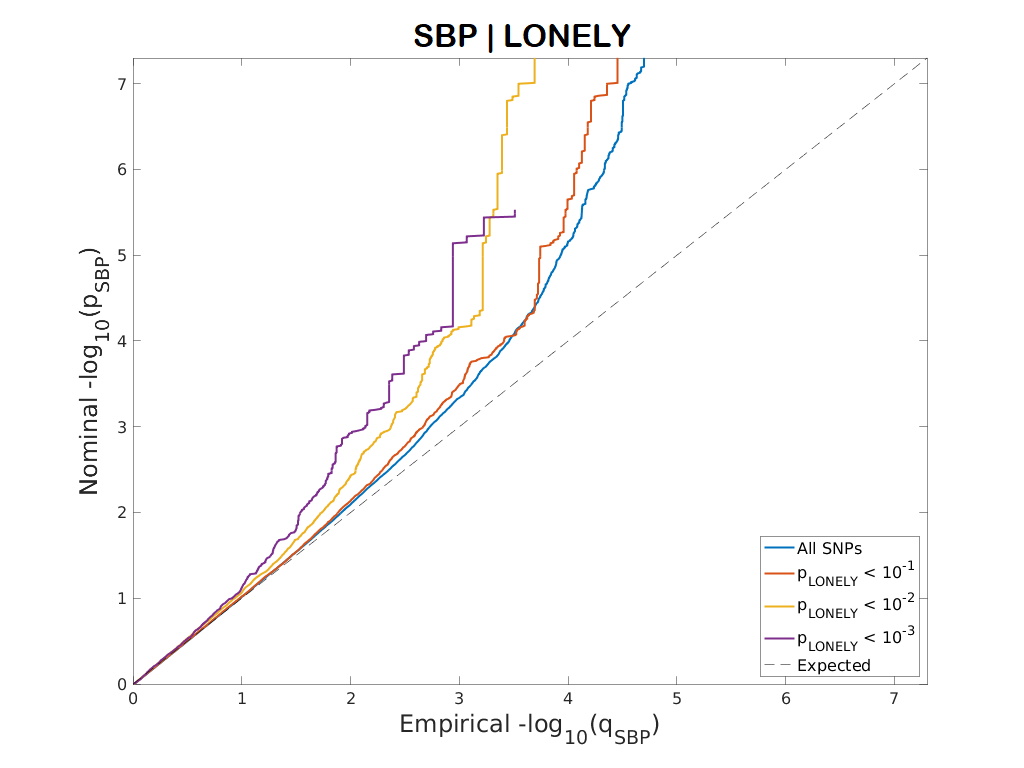


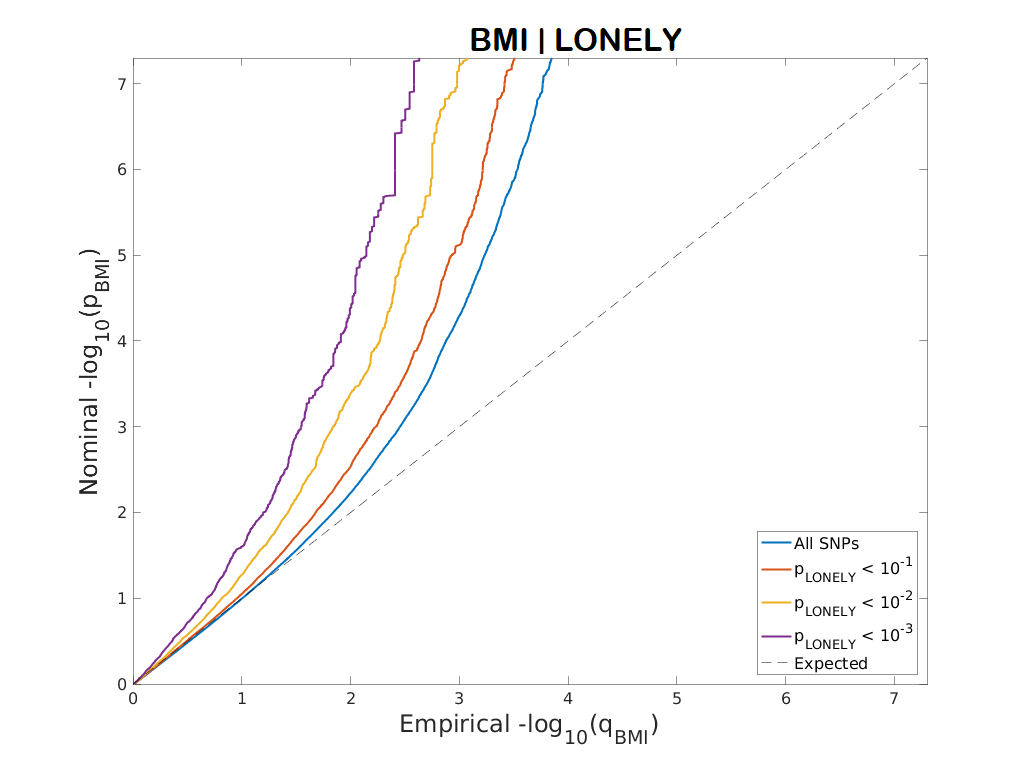

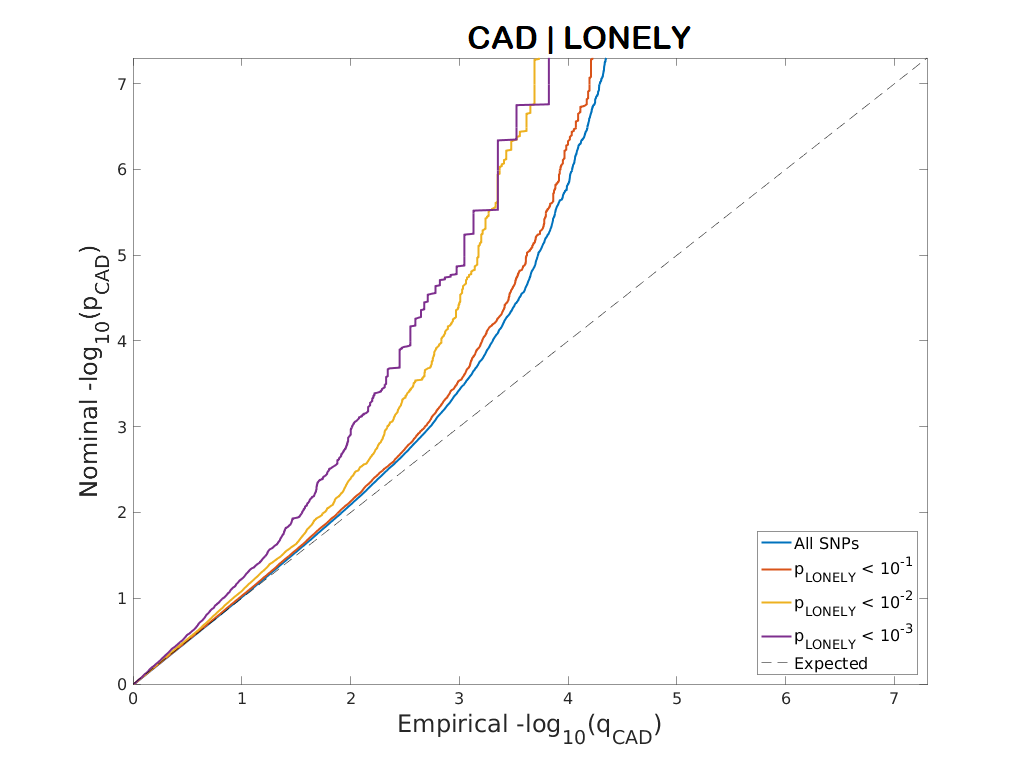

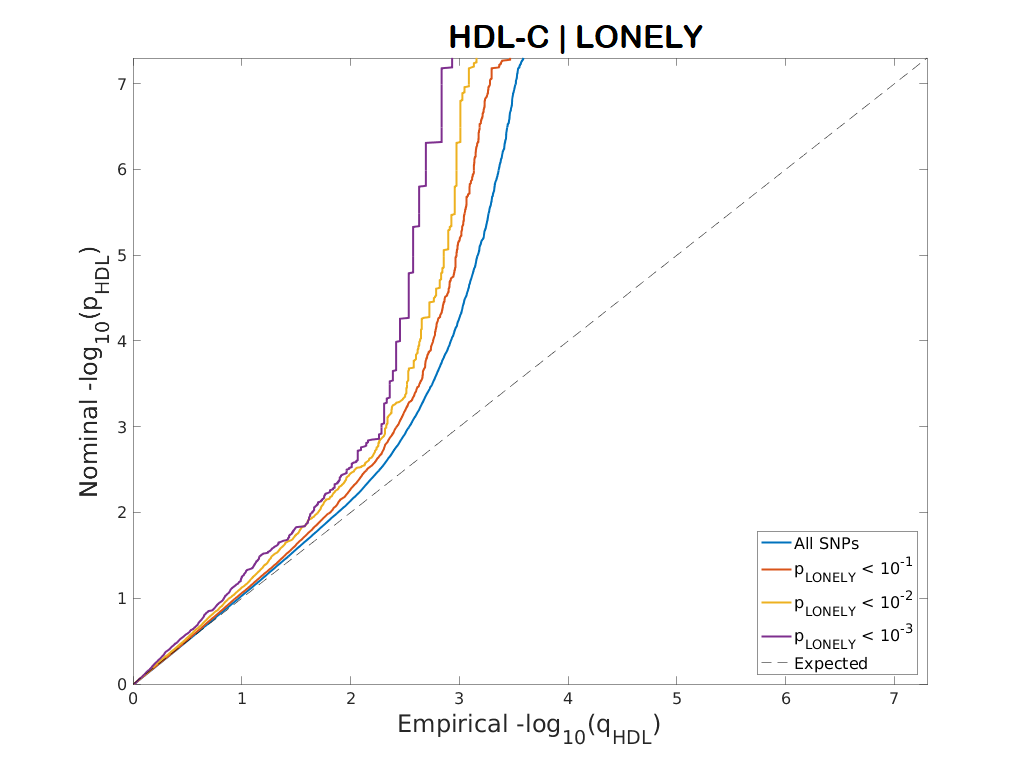

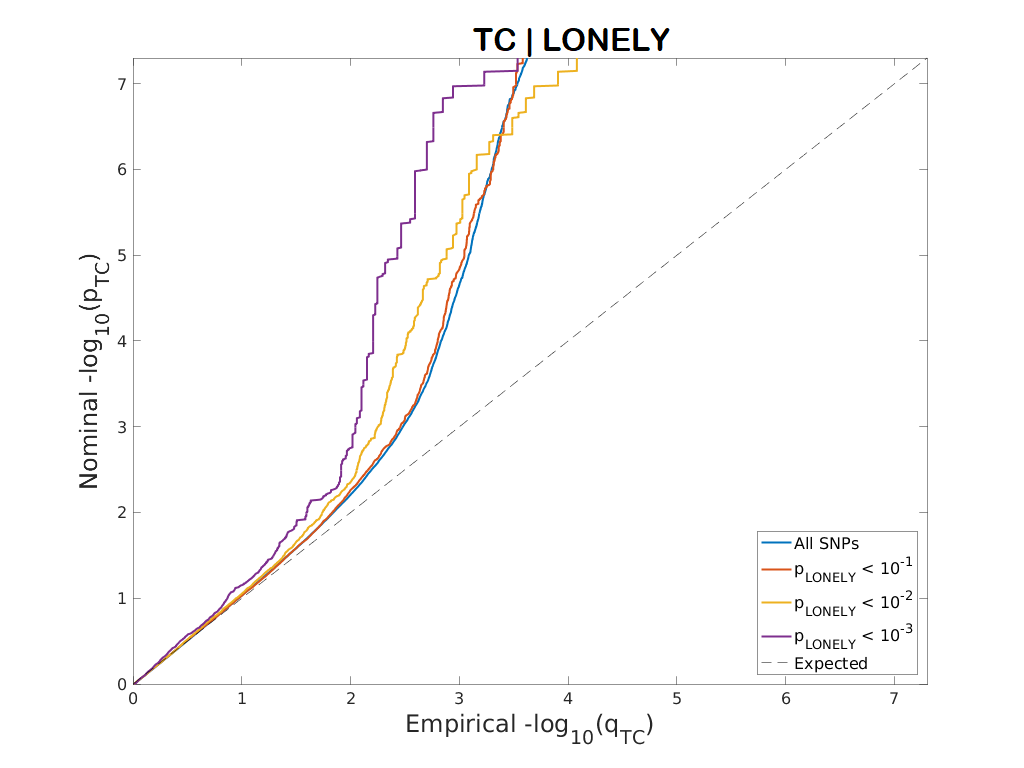


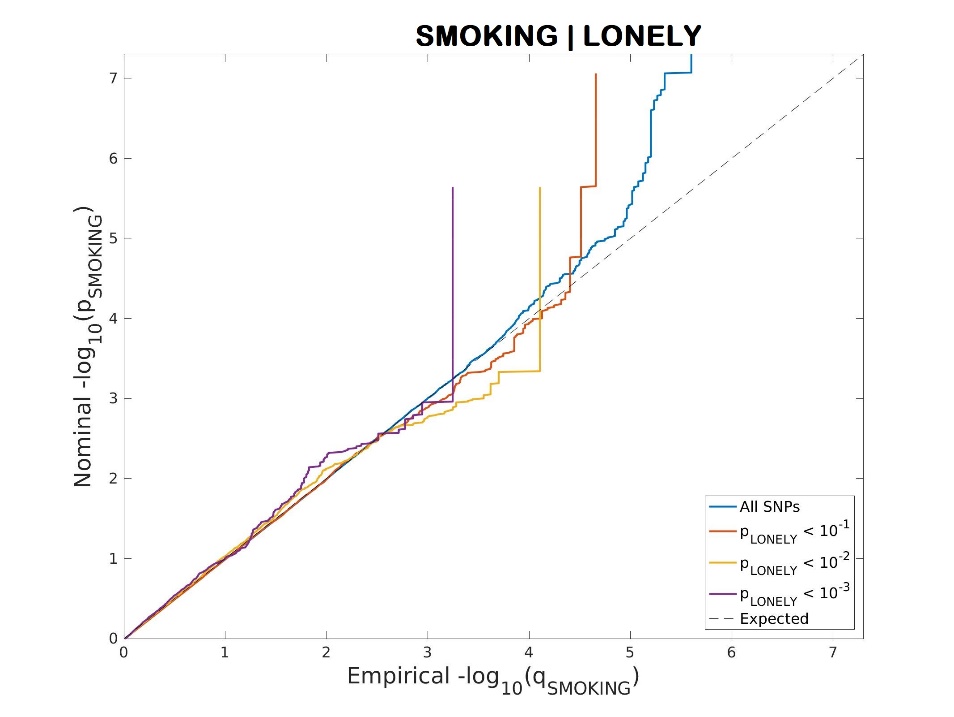

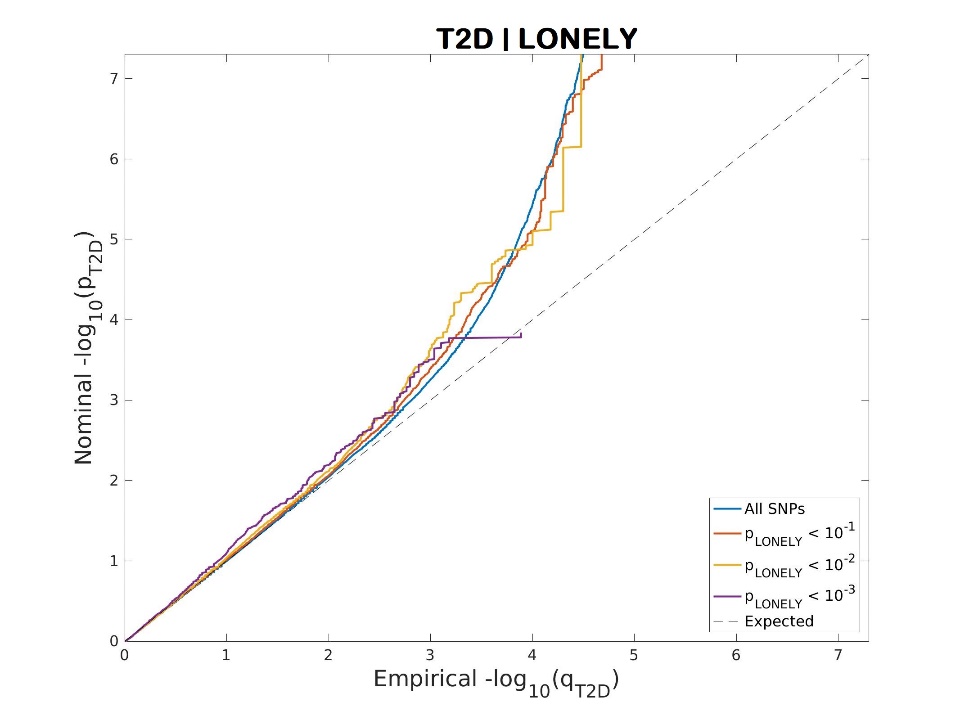


**Supplementary Figure 7.** Polygenic overlap between loneliness and CVD risk factors. Conditional Q-Q plots of nominal versus empirical −log_10_ p-values (corrected for inflation) in CVD risk factors below the standard GWAS threshold of p < 5×10^−8^ as a function of significance of association with the loneliness at the level of p < 0.1, p < 0.01, p < 0.001, respectively. The blue lines indicate all SNPs. The dashed lines indicate the null hypothesis. Abbreviations: CVD, cardiovascular disease; BMI, body mass index; CAD, coronary heart disease; HDL-C, high-density lipoprotein cholesterol; TC, total cholesterol; SBP, systolic blood pressure; DBP, diastolic blood pressure; T2D, type 2 diabetes mellitus. The conditional Q-Q plots build on the condFDR method.


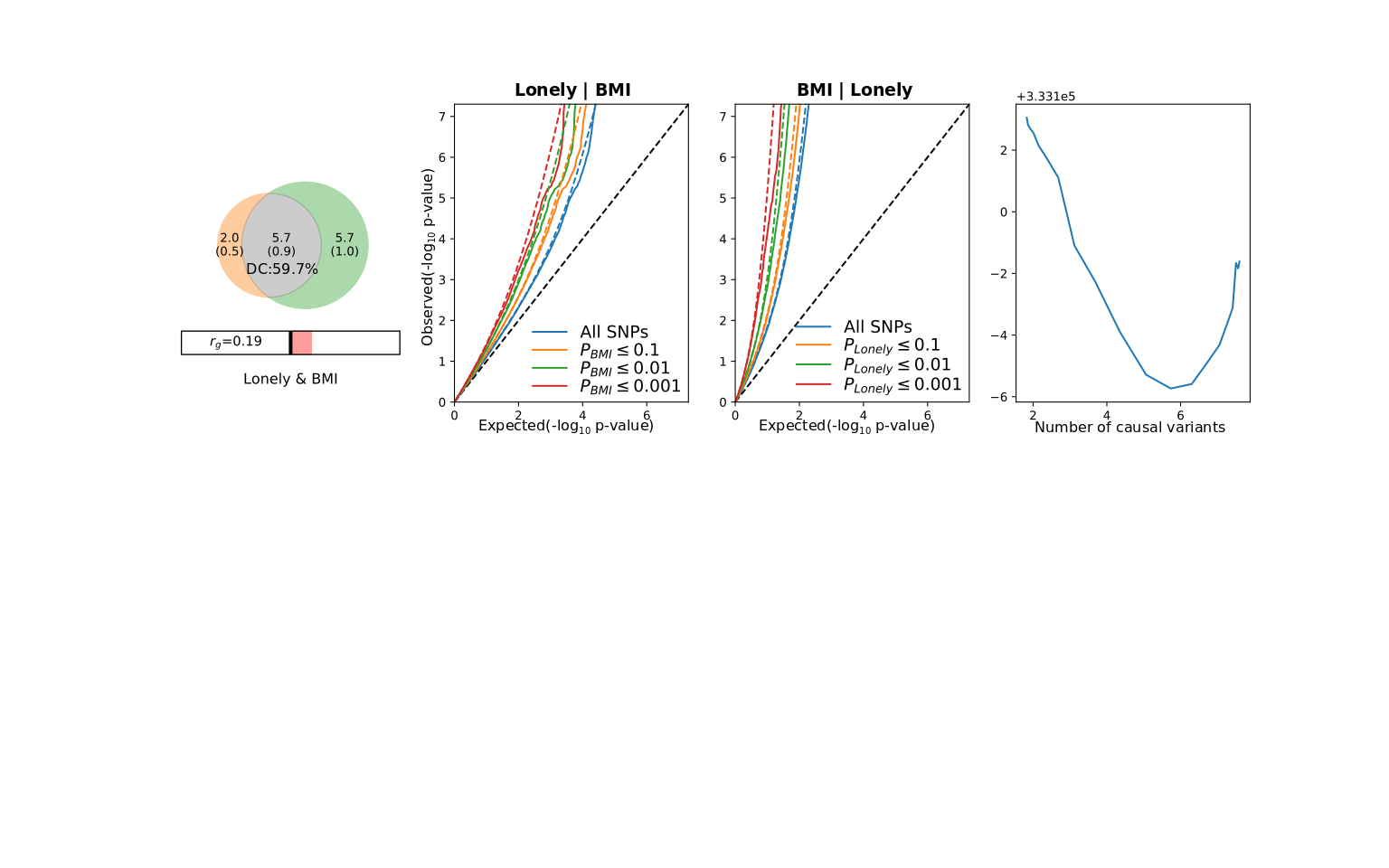


**Supplementary Figure 8.** Venn diagram, conditional Q-Q plots, and negative log-likelihood plot, respectively. Venn diagram of unique and shared polygenic components at the causal level, showing polygenic overlap (gray) between loneliness (orange) and body mass index (BMI) (green). The numbers indicate the estimated quantity of causal variants (in thousands) per component, explaining 90% of SNP heritability in each phenotype, followed by standard error, and Dice coefficient (DC) indicates the percentage of shared causal variants between the two phenotypes. Appearance of the Q-Q plot and negative log-likelihood plot are described below Supplementary Figure 3. Figures generated from MiXeR.


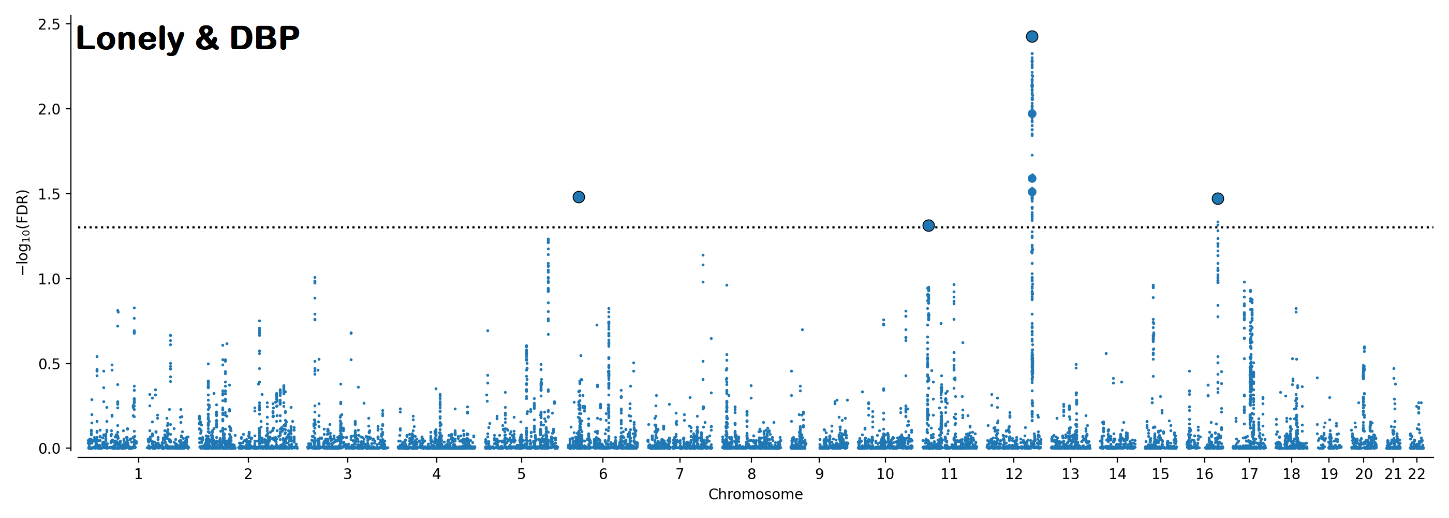

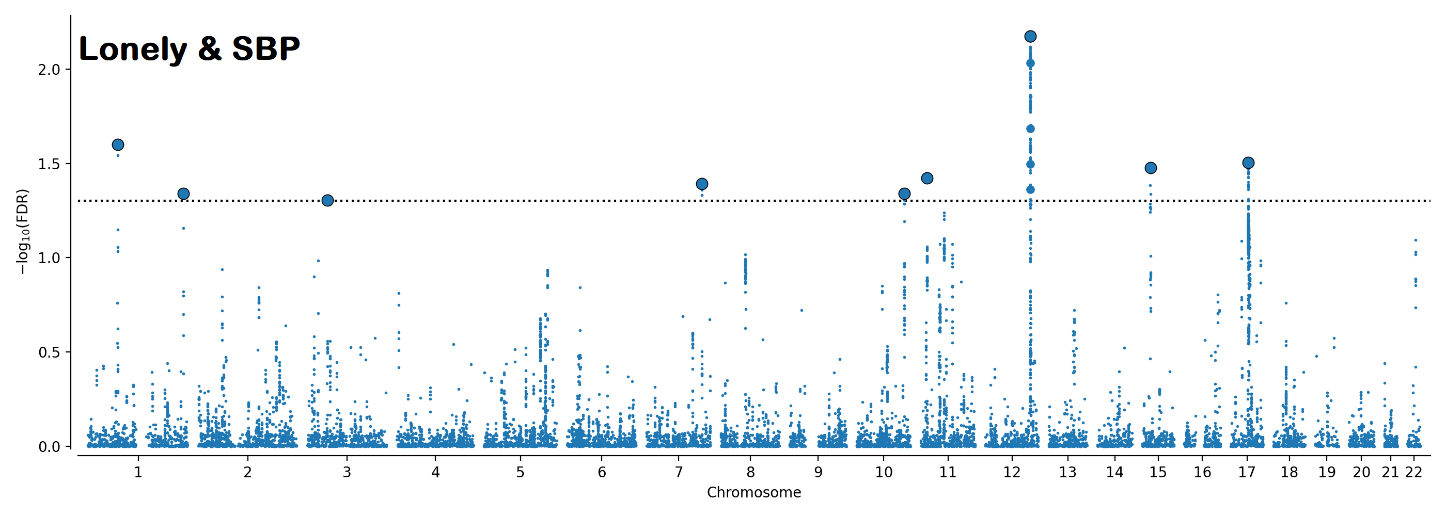


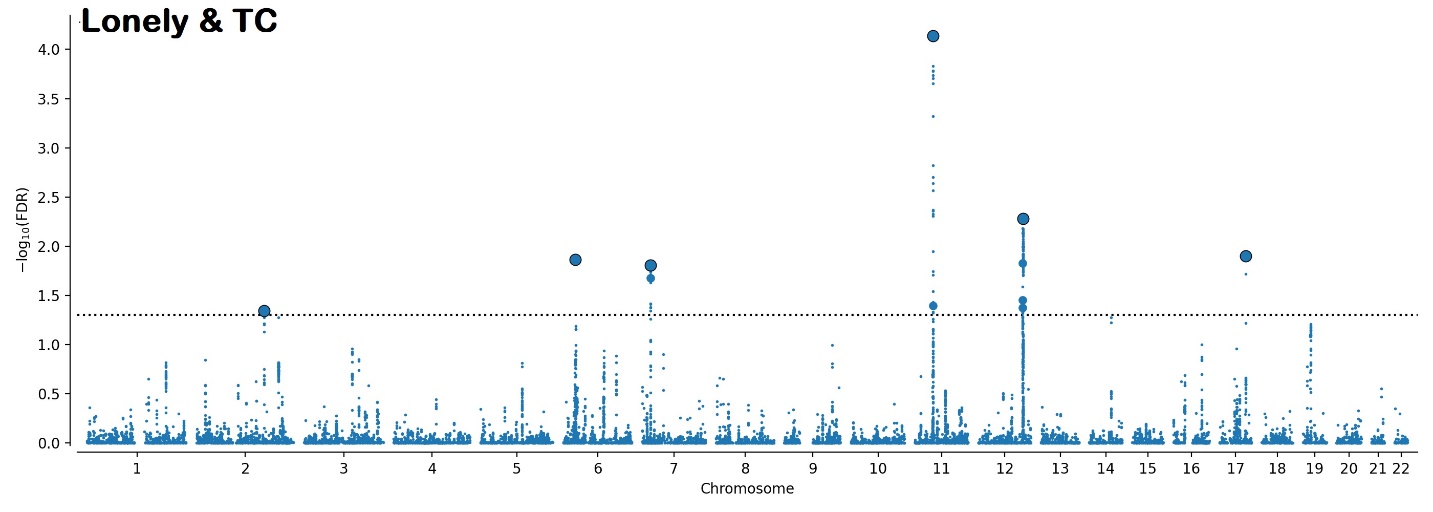

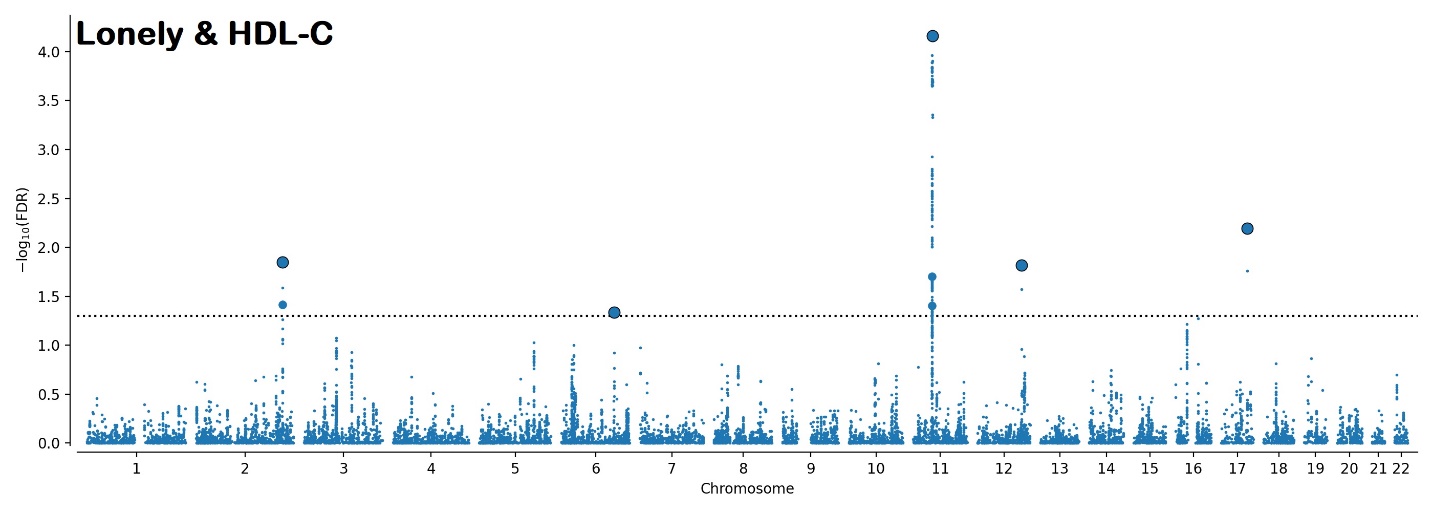


**Supplementary Figure 9.** Common genetic variants jointly associated with loneliness and CVD risk factors at conjFDR < 0.05. Manhattan plot showing the –log10 transformed conjFDR values for each SNP on the y axis and chromosomal positions along the x axis^4^. SNPs with conjFDR < 0.05 (i.e., −log10 FDR > 1.3) are shown with enlarged data points. A black circle around the enlarged data points indicates the most significant SNP in each LD block. The figure shows the localization of the ‘conjunctional loci’, and further details are provided in Supplementary Tables 21-26. Abbreviations: CVD: cardiovascular disease; TC, total cholesterol; HDL-C, high-density lipoprotein cholesterol; DBP, diastolic blood pressure; SBP, systolic blood pressure; CAD, coronary heart disease; T2D, type 2 diabetes mellitus: conjFDR, conjunctional FDR.


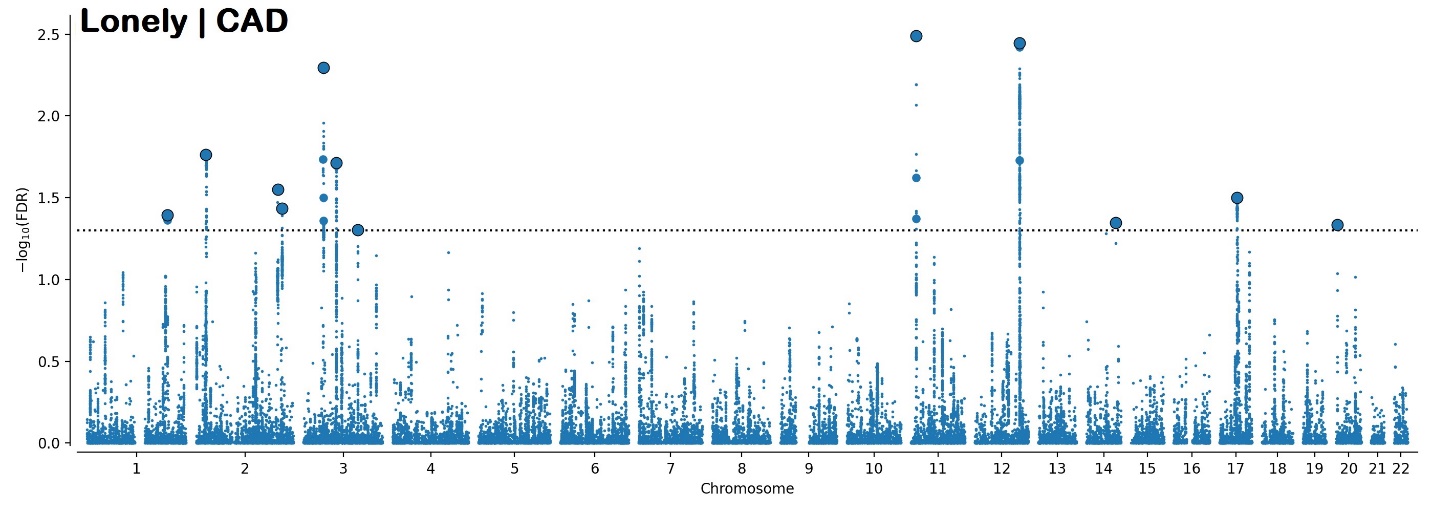

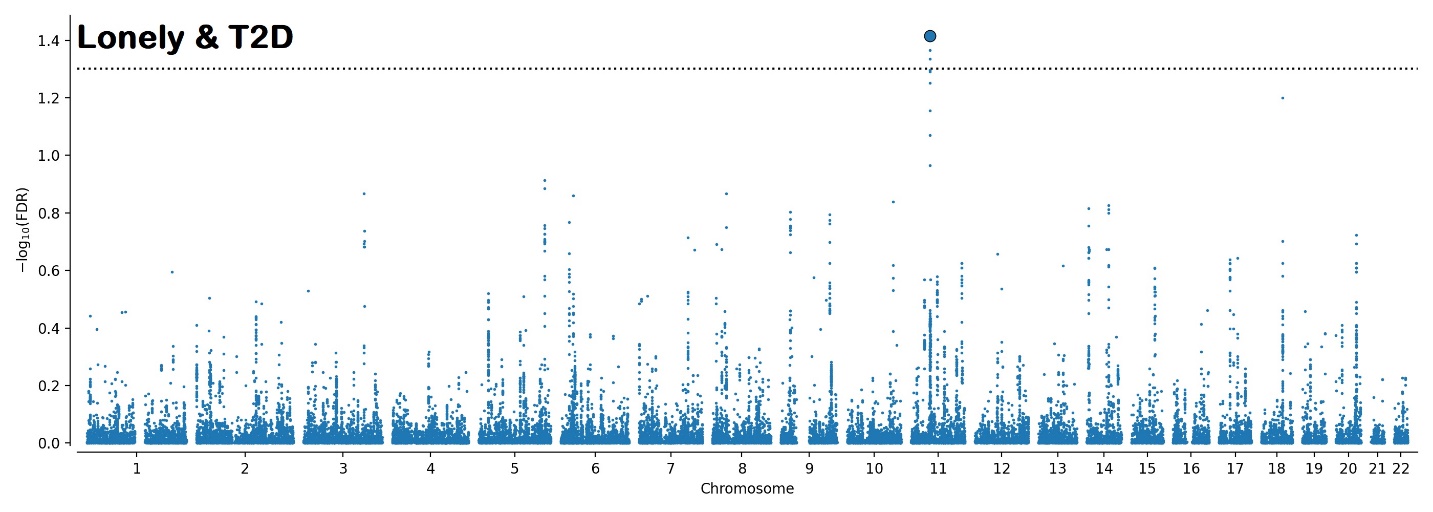


| **Supplementary Table 1. ICD-coded diagnosis in the UK Biobank** | | |
| --- | --- | --- |
| **ICD-10 diagnosis** | **Count** | **Percentage of the UK Biobank sample** |
| F20-29 Schizophrenia/schizotypal and delusional disorder | 2483 | 0.6% |
| F30 Manic episode; F31 Bipolar disorder | 245; 2123 | 0.6%^a^ |
| F32 Depressive episode;  F33 Recurrent depressive disorder;  F34 Persistent mood disorders;  F38/39 Other/Unspecified mood disorders  Major depressive disorder^c^ | 19542;  1156;  95;  93  8276 | 5.1%^b^ |
| F40-48 Neurotic, stress-related and somatoform disorders | 14914 | 3.6% |
| F50-59 Behavioural syndrome associated with physiological disturbances/physical factors | 729 | <0.2% |
| F60-69 Disorder of adult personality and behaviour | 800 | <0.2% |
| F70-99 Other behavioural and mental disorders | 1018 | 0.2% |
| Numbers of ICD-10 coded psychiatric diagnoses from the UK Biobank data field 41270. Data was available from 410 320 individuals.  ^a^This percentage includes manic episode and bipolar disorder.  ^b^This percentage includes depressive episode and depressive disorder.  ^c^The diagnostic term “major depressive disorder “ (MDD) is used in Diagnostic and Statistical Manual of Mental Disorders (DSM), not in ICD-10. The number of individuals meeting the criteria of MDD is estimated by Howard et al. 2018^38^.  ICD: International Classification of Diseases. | | |

| **Supplementary Table 2. Self-reported diagnosis in the UK Biobank sample** | | |
| --- | --- | --- |
| **Self-reported diagnosis** | **Count** | **Percentage of the UK Biobank sample** |
| Depression | 33 424 | 21.1% |
| Schizophrenia  Any other type of psychotic disorder | 157  604 | 0.1%  0.4 |
| Bipolar disorder | 837 | 0.5 |
| Anxiety, nerves or generalized anxiety | 22 036 | 14.0% |
| The numbers are retrieved from the 157 366 participants that completed the Mental Health Questionnaire and published in Davis et al. 2020^6^. | | |

| **Supplementary Table 3. Results of cross-trait analysis with the MiXeR model for loneliness, MD, SCZ, BD and BMI** | | | |
| --- | --- | --- | --- |
| **Trait1** | **Trait2** | **AIC** | |
|  |  | best vs min. overlap | best vs max. overlap |
| Loneliness | MD | -1.739 | -1.515 |
| Loneliness | SCZ | 9.231 | -1.413 |
| Loneliness | BD | 5.955 | 9.315 |
| Loneliness | BMI | 16.180 | 6.852 |
| AIC - results from Akaike information criterion, showing AIC calculated for the full versus reduced bivariate MiXeR model^14^, constrained to minimal feasible polygenic overlap (“best vs min.”) or to the complete polygenic overlap (“best vs max.”). A negative value indicates that AIC chooses reduced model, while a positive value provides an evidence for the polygenic overlap, shown in the MiXeR Venn diagram. MD: Major depression; SCZ, schizophrenia; BD, bipolar disorder; BMI, body mass index. | | | |

**SUPPLEMENTARY REFERENCES**

**E)**

**C)**

**B)**

**A)**

1. Schizophrenia Working Group of the Psychiatric Genomics Consortium. Biological insights from 108 schizophrenia-associated genetic loci. *Nature* 2014; **511**(7510)**:** 421-427.

2. Stahl EA *et al.* Genome-wide association study identifies 30 loci associated with bipolar disorder. *Nature genetics* 2019; **51**(5)**:** 793-803.

3. Wray NR *et al.* Genome-wide association analyses identify 44 risk variants and refine the genetic architecture of major depression. *Nature genetics* 2018; **50**(5)**:** 668-681.

4. Smeland OB *et al.* Discovery of shared genomic loci using the conditional false discovery rate approach. *Human Genetics* 2020; **139**(1)**:** 85-94.

5. Day FR, Ong KK, Perry JRB. Elucidating the genetic basis of social interaction and isolation. *Nature Communications* 2018; **9**(1)**:** 2457.

6. Davis KAS *et al.* Mental health in UK Biobank: development, implementation and results from an online questionnaire completed by 157 366 participants. *BJPsych Open* 2018; **4**(3)**:** 83-90.

7. Locke AE *et al.* Genetic studies of body mass index yield new insights for obesity biology. *Nature* 2015; **518**(7538)**:** 197-206.

8. Scott RA *et al.* An Expanded Genome-Wide Association Study of Type 2 Diabetes in Europeans. *Diabetes* 2017; **66**(11)**:** 2888-2902.

9. Willer CJ *et al.* Discovery and refinement of loci associated with lipid levels. *Nature genetics* 2013; **45**(11)**:** 1274-1283.

10. Ehret GB *et al.* Genetic variants in novel pathways influence blood pressure and cardiovascular disease risk. *Nature* 2011; **478**(7367)**:** 103-109.

11. Nikpay M *et al.* A comprehensive 1,000 Genomes-based genome-wide association meta-analysis of coronary artery disease. *Nature genetics* 2015; **47**(10)**:** 1121-1130.

12. Tobacco, Genetics C. Genome-wide meta-analyses identify multiple loci associated with smoking behavior. *Nature genetics* 2010; **42**(5)**:** 441-447.

13. Turcot V *et al.* Protein-altering variants associated with body mass index implicate pathways that control energy intake and expenditure in obesity. *Nature genetics* 2018; **50**(1)**:** 26-41.

14. Frei O *et al.* Bivariate causal mixture model quantifies polygenic overlap between complex traits beyond genetic correlation. *Nature communications* 2019; **10**(1)**:** 2417-2417.

15. Holland D *et al.* Beyond SNP Heritability: Polygenicity and Discoverability of Phenotypes Estimated with a Univariate Gaussian Mixture Model. *bioRxiv* 2019**:** 133132.

16. Bulik-Sullivan BK *et al.* LD Score regression distinguishes confounding from polygenicity in genome-wide association studies. *Nature Genetics* 2015; **47**(3)**:** 291-295.

17. Benjamini Y, Hochberg Y. Controlling the False Discovery Rate: A Practical and Powerful Approach to Multiple Testing. *Journal of the Royal Statistical Society. Series B (Methodological)*, vol. 57. Blackwell Publishing1995, pp 289-300.

18. Efron B. Size, power and false discovery rates. *The Annals of Statistics* 2007; **35**(4)**:** 1351–1377.

19. Purcell S *et al.* PLINK: a tool set for whole-genome association and population-based linkage analyses. *American journal of human genetics* 2007; **81**(3)**:** 559-575.

20. Schweder T, Spjotvoll E. Plots of P-Values to Evaluate Many Tests Simultaneously. *Biometrika* 1982; **69**(3)**:** 493-502.

21. Andreassen OA *et al.* Improved detection of common variants associated with schizophrenia by leveraging pleiotropy with cardiovascular-disease risk factors. *American journal of human genetics* 2013; **92**(2)**:** 197-209.

22. Schork AJ, Wang Y, Thompson WK, Dale AM, Andreassen OA. New statistical approaches exploit the polygenic architecture of schizophrenia--implications for the underlying neurobiology. *Curr Opin Neurobiol* 2016; **36:** 89-98.

23. Andreassen OA *et al.* Improved detection of common variants associated with schizophrenia and bipolar disorder using pleiotropy-informed conditional false discovery rate. *PLoS genetics* 2013; **9**(4)**:** e1003455.

24. Andreassen OA *et al.* Genetic pleiotropy between multiple sclerosis and schizophrenia but not bipolar disorder: differential involvement of immune-related gene loci. *Molecular psychiatry* 2015; **20**(2)**:** 207-214.

25. Andreassen OA, Thompson WK, Dale AM. Boosting the power of schizophrenia genetics by leveraging new statistical tools. *Schizophrenia bulletin* 2014; **40**(1)**:** 13-17.

26. Andreassen OA *et al.* Abundant genetic overlap between blood lipids and immune-mediated diseases indicates shared molecular genetic mechanisms. *PloS one* 2015; **10**(4)**:** e0123057.

27. Nichols T, Brett M, Andersson J, Wager T, Poline JB. Valid conjunction inference with the minimum statistic. *Neuroimage* 2005; **25**(3)**:** 653-660.

28. Schwartzman A, Lin X. The effect of correlation in false discovery rate estimation. *Biometrika* 2011; **98**(1)**:** 199-214.

29. Watanabe K, Taskesen E, van Bochoven A, Posthuma D. Functional mapping and annotation of genetic associations with FUMA. *Nature communications* 2017; **8**(1)**:** 1826.

30. The 1000 Genomes Project Consortium. A global reference for human genetic variation. *Nature* 2015; **526**(7571)**:** 68-74.

31. Bulik-Sullivan B *et al.* An atlas of genetic correlations across human diseases and traits. *Nature genetics* 2015; **47**(11)**:** 1236-1241.

32. Kircher M *et al.* A general framework for estimating the relative pathogenicity of human genetic variants. *Nat Genet* 2014; **46**(3)**:** 310-315.

33. Boyle AP *et al.* Annotation of functional variation in personal genomes using RegulomeDB. *Genome research* 2012; **22**(9)**:** 1790-1797.

34. Zhu Z *et al.* Integration of summary data from GWAS and eQTL studies predicts complex trait gene targets. *Nat Genet* 2016; **48**(5)**:** 481-487.

35. Kundaje A *et al.* Integrative analysis of 111 reference human epigenomes. *Nature* 2015; **518**(7539)**:** 317-330.

36. Smeland OB *et al.* Genome-wide Association Analysis of Parkinson’s Disease and Schizophrenia Reveals Shared Genetic Architecture and Identifies Novel Risk Loci. *Biological Psychiatry*.

37. Schmitt AD *et al.* A Compendium of Chromatin Contact Maps Reveals Spatially Active Regions in the Human Genome. *Cell Rep* 2016; **17**(8)**:** 2042-2059.

38. Howard DM *et al.* Genome-wide association study of depression phenotypes in UK Biobank identifies variants in excitatory synaptic pathways. *Nature Communications* 2018; **9**(1)**:** 1470.
